# Implementation of UNICEF and WHO's care for child development package: Lessons from a global review and key informant interviews

**DOI:** 10.3389/fpubh.2023.1140843

**Published:** 2023-02-16

**Authors:** Marilyn N. Ahun, Frances Aboud, Claire Wamboldt, Aisha K. Yousafzai

**Affiliations:** ^1^Department of Global Health and Population, Harvard T.H. Chan School of Public Health, Boston, MA, United States; ^2^Department of Psychology, McGill University, Montreal, QC, Canada

**Keywords:** care for child development, implementation, nurturing care, early child development (ECD), program evaluation

## Abstract

**Introduction:**

In the last decade, there has been increased global policy and program momentum to promote early childhood development. The Care for Child Development (CCD) package, developed by UNICEF and the WHO, is a key tool responding to the global demand. The CCD package comprises two age-specific evidence-based recommendations for caregivers to 1) play and communicate and 2) responsively interact with their children (0–5 years) and was designed to be integrated within existing services to strengthen nurturing care for child development. The aim of this report was to provide an up-to-date global review of the implementation and evaluation of the CCD package.

**Methods:**

In addition to a systematic review of 55 reports, we interviewed 23 key informants (including UNICEF and WHO personnel) to better understand the implementation of CCD.

**Results:**

The CCD package has been or is being implemented in 54 low- and middle-income countries and territories, and it has been integrated into government services across the health, social, and education sectors in 26 countries. Across these contexts, CCD has been adapted in three primary ways: 1) translations of CCD materials (mostly counseling cards) into local language(s), 2) adaptations of CCD materials for the local context, vulnerable children, or a humanitarian/emergency setting (e.g., including local play activities, using activities that are better suited to children with visual impairments), and 3) substantive modifications to the content of CCD materials (e.g., expansion of play and communication activities, addition of new themes, creation of a structured curriculum). While there is promising evidence and examples of good implementation practice, there has been mixed experience about implementation of CCD with respect to adaptation, training, supervision, integration into existing services, and monitoring implementation fidelity and quality. For example, many users of CCD found difficulties with training the workforce, garnering buy-in from governments, and ensuring benefits for families, among others.

**Discussion:**

Additional knowledge on how to improve the effectiveness, implementation fidelity and quality, and acceptance of CCD is needed. Based on the findings of the review we make recommendations for future efforts to implement CCD at-scale.

## 1. Introduction

The early childhood environment plays a significant role in child development. Evidence across health and social science disciplines shows that the origins of adult disease and wellbeing lie in the developmental processes that occur during early childhood (1–4). Ensuring that children grow up in a stable environment that nurtures and promotes their health, nutritional, and developmental needs while protecting them from threats and providing them with interactions that are responsive, emotionally supportive, and stimulating is therefore a key priority for promoting health across the lifespan (1, 3, 5–7). The Nurturing Care Framework [NCF] (8) advocates services which address these holistic components (*health, nutrition, responsive caregiving, security and safety*, and *early learning*) of nurturing care in multisectoral systems. Early childhood interventions that target one or more of the NCF components are known to effectively promote nurturing care practices and child development (1, 6). However, there remain important gaps in our understanding of how to scale-up and integrate these interventions into existing services while maintaining quality programming and implementation (3). The objective of this paper is to provide a review of the implementation and evaluation of a specific early childhood parenting skills package, namely UNICEF and the World Health Organization's (WHO) Care for Child Development (9), across contexts and to present recommendations to improve its roll-out.

The Care for Child Development (CCD) package was developed by UNICEF and the WHO to promote nurturing care and child development for children aged 0–5 years through integration in existing services, primarily in the health sector (9). Specifically, the package aims to build skills of providers to support caregivers in responsive caregiving and early learning activities, and improve caregiver-child interactions through responsive play and communication. CCD was adapted from UNICEF and the WHO's Integrated Management of Childhood Illness (IMCI) strategy, which sought to address the common causes of childhood mortality in low- and middle-income countries (10, 11). Although the IMCI strategy helped to reduce rates of child mortality, there was concern that the developmental needs of the majority of children who survived were not being met (10). The WHO therefore commissioned reviews of effective early childhood interventions to address this gap (12). These reviews informed the development of the CCD package, which provides guidance to delivery agents on how to help caregivers interact responsively with their young children and provide opportunities for early learning (11, 13). It is important to note that CCD was not intended to be implemented as a standalone package but integrated within existing services to strengthen care for child development.

The CCD package consists of two age-specific evidence-based recommendations for caregivers to (1) play and communicate with their children in a (2) responsive^1^ manner. These recommendations are designed to change child and caregiver outcomes over time. In the short-term, expected changes include an increase in the number of available play materials a child can engage with in the home and the quality and quantity of responsive stimulation (i.e., playing, talking, singing, etc.) a caregiver provides. This can lead to improvements in the quality of responsive caregiver-child interactions and in children's developmental outcomes. To enable delivery agents to administer the CCD package, they are equipped with a participant manual which includes an overview of child development and the importance of nurturing care, some age-specific recommendations for play and communication, counseling cards^2^ to use as a visual aid when discussing these recommendations with caregivers, and a checklist^3^ to help them identify caregivers' care practices (9). UNICEF and the WHO also provide Facilitator Notes^4^, a Guide for Clinical Practice, and a Framework for Monitoring and Evaluating intended to support the training of providers (9).

To date, there have been two reports summarizing the implementation and evaluation of interventions and/or services (henceforth collectively referred to as services) that have incorporated the CCD package (11, 13). The extent to which the CCD package has been incorporated varies greatly across these programs, ranging from those that broadly follow the recommendations of CCD to include developmentally stimulating opportunities and responsive caregiving interactions (henceforth referred to as CCD-informed), to those that use the participant manual and other CCD materials to guide implementation, here called CCD-based. The first report by Lucas et al. (11) focused on CCD-based services. They found that 23 sites had integrated the CCD package within a range of government and non-government services including child survival and health, nutrition rehabilitation, infant care and early education, services to families with developmentally disabled children, and a conditional cash transfer program (11). Despite the implementation of the CCD package in various sites, there were only three sites (China, Pakistan, and Turkey) that published evaluations of the impact of CCD on child and caregiver outcomes (15–17). Overall, these studies found improvements in children's cognitive and language development and parenting practices such as responsive caregiver-child interactions and greater availability of learning materials in the home. However, there was variability in how the CCD package was implemented (2 clinic visits in China and Turkey compared to 40 group sessions and home visits in Pakistan) and one study found that higher quality training and more regular supervision were needed to strengthen implementation (11). The second report (13), a scoping review, included CCD-based and CCD-informed services that used messages regarding play, communication, and responsiveness in their intervention content in addition to ones that used the CCD package to counsel caregivers. The report found that many services had been evaluated and shown to be effective in improving child and caregiver outcomes. However, few of them reported information on implementation processes, except for information about curriculum and workforce training, thus limiting our understanding of how they work. Scale and sustainability information was also lacking.

The objective of this report was to examine the implementation information emerging from both CCD-based and CCD-informed reports by providing a systematic review of CCD to clarify how it is being implemented in different contexts around the world and how it benefits caregivers and children. Specifically, our objectives are to:

i Identify and summarize peer-reviewed and gray literature reports of CCD implementation, including program content and adaptations, delivery modalities, characteristics of delivery agents, monitoring of implementation processes, characteristics of intervention beneficiaries, and evaluation of intervention outcomes;
ii Describe the extent to which CCD has been integrated into government services;
iii Identify barriers and facilitators to CCD implementation.

## 2. Methods

This report consists of a mixed-methods review of CCD. First, we conducted a systematic review of scientific and gray literature reports concerning the implementation and/or evaluation of the CCD package. Secondly, we supplemented this information by conducting in-depth interviews with key informants (i.e., individuals who have been involved in the development, implementation, or evaluation) of CCD. These methods are further described below.

Reports were identified using both electronic and manual searches. Electronic searches were conducted in PubMed and Global Health Ovid using a search strategy informed by search terms and keywords used in prior systematic reviews of early childhood services (18, 19). Reference lists of relevant studies were scanned for any additional reports that may have been missed. Specifically, we searched the reference lists of two previous CCD reports (11, 13). Additional reports were identified through the key informant interviews and discussions with early childhood development (ECD) experts who are familiar with the CCD package and staff in the ECD Section at UNICEF Headquarters and WHO Headquarters. The sole inclusion criterion was that the report included data on the implementation (e.g., delivery modality and dosage, adaptations to CCD, behavior change techniques, characteristics of program beneficiaries and delivery agents, training, monitoring, and supervision of delivery agents, integration of CCD into existing services) or evaluation (e.g., study design, sample size, impact of CCD on child development and/or caregiving) of a program that was informed by or based on the CCD package. Data on the evaluation and implementation of CCD were extracted according to the Consolidated Advice on Reporting ECD Implementation Research (CARE; (20)) and Consolidated Standards for Reporting Trials [CONSORT; (21)] guidelines.

The review was complemented by in-depth interviews with individuals who: (1) have been directly involved in the implementation (including training, monitoring, or supervising delivery agents) or evaluation of CCD; (2) have been involved in the development of the CCD package and/or delivery of Master Training workshops; and/or (3) have considered the CCD package for use in a parenting intervention. These individuals were identified during the review (i.e., authors of reports or individuals identified as being involved in implementation or evaluation of CCD in reports) and from discussions with ECD experts and staff from the ECD Section at UNICEF Headquarters and WHO Headquarters. A semi-structured topic guide was developed to include questions about implementation (including training, delivery, monitoring), evaluation, the challenges and successes of the implementation or evaluation processes, and their recommendations for improving the uptake and implementation of CCD (see Appendix 1, Online Supplementary material).

Twenty-four informants were identified and contacted for the first round of key informant interviews. These informants—specifically those who had implemented or evaluated CCD or had consulted the CCD package when developing an intervention—were identified during the review (*n* = 8) and through discussions with ECD experts (*n* = 14) and the ECD section at UNICEF Headquarters (*n* = 2). Of the 24 informants contacted, 19 agreed to be interviewed and the remaining 5 did not respond to our initial and follow-up emails. The first round of interviews was conducted by the first author of this review (MNA) with support from the second and third authors, from 19th January to 24th February 2022 using a semi-structured topic guide (see Appendix 1 for topic guide). The interviews were conducted virtually in English on a password-protected Zoom call that lasted an hour and was recorded to facilitate data analysis. Data were analyzed between 8*th* February and 4^th^ March 2022 using thematic content analysis. The first author (MNA) initially reviewed notes from 4 interviews and developed an analysis grid of key themes, based on inductively identified codes (see Appendix 1 for analysis grid). This analysis grid was iteratively refined in a meeting with all authors who then used the final version of the analysis grid to independently analyze 3 to 6 interviews each.

Data from the first round of interviews informed the preparation of a semi-structured topic guide for the second round of interviews, which included four key informants [i.e., UNICEF (*n* = 2) and WHO (*n* = 1) staff and a CCD Master Trainer] involved in the development or management of CCD implementation globally. This guide included questions on informants' objectives for the future of CCD and their reflections on the issues raised by informants from the first round of interviews (see Appendix 1). These interviews were also conducted virtually by MNA. They lasted ~1.5 hours and were held on 3rd and 8th March 2022. Data from these interviews were used to complement the themes identified in the first round of reviews and to inform the discussion. Overall, we interviewed 23 informants (19 in the first round of interviews and 4 in the second). Participants provided informed consent and ethics approval was obtained from McGill University's Institutional Review Board (#21-11-029). The results are reported according to the Consolidated Criteria for Reporting Qualitative Research (22).

## 3. Results

**Figure 1:**
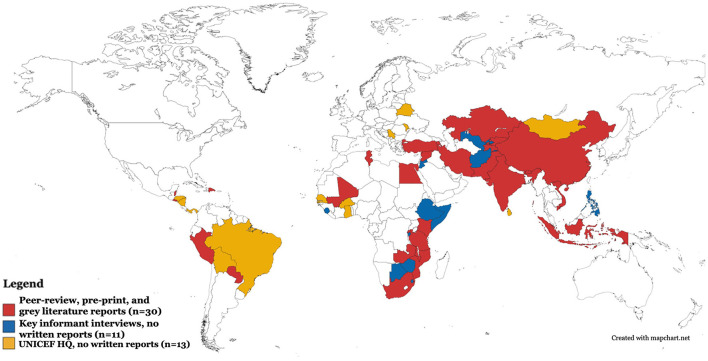
Map of countries and territories reporting use of CCD package.

We identified a total of 28 documents (26 peer-reviewed and 2 gray literature reports) describing the training, implementation, or evaluation of the CCD package in 17 services across 14 low- and middle-income countries through the review. Through discussions with ECD experts (*n* = 11) and the ECD Section at UNICEF Headquarters (*n* = 2), and the key informant interviews (*n* = 14), we identified 27 additional documents (8 peer-reviewed, 2 pre-print, and 17 gray literature reports) describing 41 additional services in 21 countries and territories, including 16 countries and territories for which we did not find reports in the review. We also learned about the implementation of CCD in 24 additional countries from the key informant interviews (*n* = 11) and documents shared by UNICEF Headquarters (*n* = 13) which did not meet our inclusion criterion (i.e., they did not include data on the implementation or evaluation of a CCD intervention or service). These UNICEF documents included the Latin America and Caribbean Regional Office's (LACRO) CCD roll-out guide and a survey of countries reporting whether CCD was used in any in-country parenting programs. All countries and territories that are reported to use CCD—including those for which a written report is not available—are shown in Figure 1 and listed by UNICEF world regions in Panel 1. Countries for which we have a written report (peer-reviewed, pre-print, or gray literature) describing CCD implementation or evaluation are shown in red and those for which we do not have a written report are shown in blue (key informant interviews) and yellow (UNICEF Headquarters) according to the source of information.

**PANEL 1:** List of countries and territories reporting use of CCD package by UNICEF world regions.

**East Asia and Pacific** • ^*^China • ^*^Indonesia • ^*^Mongolia • ^*^Philippines • ^*^Vietnam **Eastern Europe and Central Asia** • ^*^Armenia • ^*^Belarus • ^*^Kazakhstan • ^*^Kyrgyzstan • ^*^Moldova • ^*^Serbia • ^*^Tajikistan • ^*^Turkey • ^*^Uzbekistan **Eastern and Southern Africa** • ^*^Botswana • ^*^Burundi • ^*^Eswatini • ^*^Ethiopia • ^*^Kenya	**Eastern and Southern Africa (cont'd)** • ^*^Malawi • ^*^Mozambique • ^*^Rwanda • ^*^Somalia • ^*^South Africa • ^*^Tanzania • ^*^Uganda • ^*^Zambia • ^*^Zimbabwe **Latin America and Caribbean** • ^*^Anguilla (territory) • ^*^Belize • ^*^Bolivia • ^*^Brazil • ^*^Dominican Republic • ^*^El Salvador • ^*^Honduras • ^*^Nicaragua • ^*^Panama • ^*^Paraguay • ^*^Peru	**Middle East and North Africa** • ^*^Egypt • ^*^Iran • ^*^Jordan • ^*^Syria • ^*^Tunisia **South Asia** • ^*^Afghanistan • ^*^Bhutan • ^*^India • ^*^Pakistan • ^*^Sri Lanka **West and Central Africa** • ^*^Burkina Faso • ^*^Ghana • ^*^Mali • ^*^Senegal • ^*^Sierra Leone

^*^Peer-review, pre-print, and gray literature reports (n = 30).

^*^Key informant interviews, no written reports (n = 11).

^*^UNICEF HQ, no written reports (n = 13).

**Figure 2:**
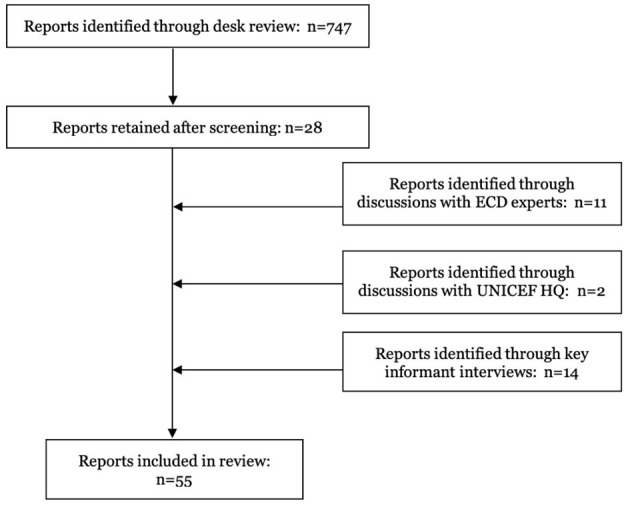
Flow diagram showing the selection procedure of peer-reviewed, pre-print, and gray literature reports included in this review.

The present review focuses on the data obtained from written reports of CCD implementation, training, and evaluation and thus summarizes information across 58 services in 30 countries and territories as described in 55 documents (34 peer-reviewed, 2 pre-print, and 19 gray literature reports) published between 2006 and 2022. A flow diagram of the procedure for including studies is in Figure 2. Where available, information on the content and structure of services (Supplementary Table S1), their settings and beneficiaries (Supplementary Table S2), characteristics and training of delivery agents (Supplementary Table S3), and evaluation of their impact on child and caregiver outcomes (Supplementary Table S4) were extracted into tables which can be found in Appendix 2 (Online Supplementary material). First, we describe the extracted data in Supplementary Tables S1–S4 and provide a general summary of our findings. We then summarize results from the thematic content analysis of the key informant interviews, including informants' perception and understanding of the CCD package, its strengths and weaknesses, and suggestions for how UNICEF and the WHO can better support CCD implementation and evaluation.

### 3.1. Summary of systematic review

The content and structure of services are described in Supplementary Table S1 (which can be found in Appendix 2). This review includes services which varied greatly in the extent to which they incorporated the CCD package. For clarity, these are defined as being either CCD-informed (i.e., authors claimed to use some features of CCD along with their own or other parenting programs) or CCD-based (i.e., using the CCD package as the sole reference for developing a service). An example of a CCD-based service is described by (15) [Turkey], where pediatricians used the counseling cards to recommend play and communication activities and identified care practices using the CCD observation checklist for caregivers. In contrast, Rockers et al., [(23), Zambia] implemented a CCD-informed intervention, where CCD was one of a handful of ECD and parenting packages that researchers consulted and used to develop the intervention curriculum. Most services (*n* = 46) were CCD-based. Although all of these services were based on and formally defined as CCD, among those that reported the relevant data, there was great variation in the delivery modalities used (clinic visits [*n* = 8], home visits [*n* = 8], group sessions [*n* = 7], some combination of clinic and home visits and group sessions [*n* = 20]), the intensity of contacts between delivery agents and program beneficiaries (ranging from one 5-min session to 40 fortnightly sessions over two years), and the use of job aids (26 CCD-based services reported using job aids in sessions). A small number of CCD-based services (*n* = 3) also created playboxes, filled with homemade age-appropriate play objects and reading materials, in clinical settings to encourage caregivers to play with their children while waiting for health services.

There was also variation in the content of CCD-based services, including the extent to which adaptations, if any, were made to the CCD package. Three kinds of adaptations were described across reports: (1) translations of CCD materials into the local language(s), (2) substantive modifications to the content of CCD materials, and (3) adaptations of CCD materials for the local context, vulnerable children, or a humanitarian/emergency setting. Examples of translations of and substantive modifications to CCD materials are described below, while examples of adaptations for local contexts are provided in the next section on settings and beneficiaries of services using CCD.

Counseling cards were translated into a variety of local languages including Arabic, Chinese, Farsi, French, Guaraní, Gujarati, Hindi, Javanese, Kakwa, Kannada, Kinyarwanda, Kiswahili, Luganda, Marathi, Spanish, and Vietnamese. Some studies simply made cultural adaptations to CCD materials by, for example, using traditional games as play activities. Others made substantive modifications by providing more specific recommendations on how to engage in responsive play and communication activities and creating a structured curriculum to guide delivery agents in their interactions with caregivers. For example, seven studies explicitly reported making substantive modifications to the play and communication activities of the CCD package ((24) [Indonesia]; (25) [Mozambique], (26) [Tanzania]; (27) [Malawi]; (28) [Kenya]; (29) [India and Pakistan]; (17) [Pakistan]^5^). In Malawi, this consisted of modifying play and communication activities to focus on touch, hearing, and other senses for children with visual impairments (27). Other substantive modifications consisted of expanding the content of sessions to include additional topics such as mental health and emotional development (e.g., (30) [Turkey]), positive discipline (e.g., (26) [Tanzania], (17) [Pakistan]), and caregiver mental health (e.g., (31) [India], (17) [Pakistan]) among other topics.

Of all the services reviewed, twenty of them reported whether CCD had been delivered alongside (i.e., bundled with) another package. CCD was primarily bundled with health (e.g., the WHO's IMCI strategy: (32) [Kenya]; (33) [Uganda]; (34) [Malawi]; (35) [Tanzania]; (36) [Egypt]) and nutrition education ((34) [Malawi]; (26) [Tanzania]; (17) [Pakistan]). Only three services delivered CCD alongside a package that targets the mental health and psychosocial wellbeing of caregivers [i.e., (37) the Thinking Healthy Program; (38) [Vietnam]; (29) [India and Pakistan] and Behavioral Activation Therapy: (39) [South Africa]].

Services also used a variety of techniques to encourage behavior change in caregivers. The behavior change techniques outlined in the CCD Participant Manual are print media (i.e., use of pamphlets, posters, flipcharts, or other forms of print media to convey messages), provision/creation of materials (e.g., play objects), self-performance (i.e., having caregivers practice behaviors with their child followed by coaching and feedback from a delivery agent), and problem-solving. Most services included in this review reported using some of these behavior change techniques, and some (e.g., (15) [Turkey]; (25) [Mozambique]; see Supplementary Table S1 for additional examples) used additional techniques such as other-performance (i.e., caregivers watching delivery agents demonstrate/model a behavior with the child) and social support (i.e., leveraging beneficiaries' relationships with family and community members as a source of support to facilitate behavior change). The most common behavior change technique used was self-performance, followed by the provision of materials and the use of print media. The least frequently used techniques were audio-visual media, social support from community members, and problem-solving. The total number of behavior change techniques used in a single service ranged from one to eight.

Supplementary Table S2 provides information on the setting and beneficiaries of CCD-based and CCD-informed services (see Appendix 2). Services were conducted in both rural and urban areas, with the majority taking place in rural areas. The primary beneficiaries of most services were children < 5 years old (many services specifically targeted 0–3-year-olds) and their mothers, although some services primarily focused on the primary caregiver. These beneficiaries mostly came from low socioeconomic backgrounds. Nine studies specifically targeted vulnerable groups: children born prematurely or with low birth weight ((40) [Dominican Republic]), children with visual impairments ((27) [Malawi]), children with other physical and/or cognitive disabilities ((40) [Dominican Republic]; (41) [El Salvador], (42) [Peru]; (31) [India]), children of HIV+ mothers ((43) [Malawi]; (28) [Kenya]), children of incarcerated mothers ((44) [Paraguay]), and children of HIV+ mothers experiencing antenatal depressive symptoms ((39) [South Africa]).

With respect to vulnerable settings, both CCD-based and CCD-informed services were implemented in conflict-affected areas ((45) [Syria]), during the Zika pandemic ((40) [Dominican Republic]), and as part of the emergency response to Hurricane Irma ((46) [Belize and Anguilla]). For example, within the framework of the response to the Zika pandemic in the Dominican Republic, delivery agents from a community-based organization who were conducting Zika awareness campaigns and making home visits to families affected by the virus were trained to incorporate CCD in these services ((40) [Dominican Republic]). A handful of studies also described changes to service delivery for the COVID-19 context ((32) [Kenya]; (33) [Uganda]; (47) [Mozambique]; (26) [Tanzania]; (40) [Dominican Republic]). Changes included moving service delivery to a digital platform, reducing the frequency and length of contacts between delivery agents and caregivers, and switching from a group-based delivery modality to home visits where delivery agents and caregivers respected COVID-19 mitigation protocols (e.g., social distancing, handwashing, meeting outdoors).

In addition to the primary beneficiary, some services (*n* = 20) actively engaged other caregivers in the child's life, mostly their fathers and grandmothers. In terms of program reach, the handful of documents (*n* = 8) reporting this information indicated that 30–99% of targeted caregivers participated in the described service. Reports that assessed beneficiaries' program acceptance (*n* = 20) found that overall caregivers were satisfied with service content and delivery, and they reported increased engagement in play and communication activities with their children as a result of the service. They also highlighted some issues. For example, some caregivers stated that they would have liked a better explanation of the service's purpose ((48) [Mozambique]) and to have more explicit and repetitive messages on why specific activities are important for children's development ((43) [Malawi]). Two of the reports ((24) [Indonesia]; (30) [Turkey]) described the training of delivery agents in the CCD package and one of them reported data on delivery agents' program acceptance. General practitioners and nurse-midwives in Turkey appreciated the content and materials of the CCD package and felt that the training improved their ability to engage caregivers in ECD counseling (30). They also noted some programmatic issues, including that the training focused on improving competence and knowledge rather than developing a comprehensive program that incorporated ECD into health care delivery.

Further information on the characteristics and training of delivery agents are reported in Supplementary Table S3 (see Appendix 2). Delivery agents had varying levels of education (e.g., some or completed secondary schooling, bachelor's degree, medical degree) and came from different sectors including health (e.g., nurse-midwives, pediatricians, community health workers, health assistants), education (e.g., kindergarten teachers), and social (e.g., social workers, community-based rehabilitation workers) sectors, or from the general community (e.g., community-based volunteers, caregivers). Thirty-nine studies reported whether the CCD package had been integrated into an existing government service or delivered as a separate service. All but five of these reported that CCD had been integrated into existing services in the health (Bhutan, Egypt, China, Kazakhstan, Kyrgyzstan, India, Indonesia, Malawi, Mozambique, Pakistan, Rwanda, Syria, Tajikistan, Turkey) and social (Vietnam) sectors. Nine of these reports indicated that CCD-based and CCD-informed services had been integrated into services across multiple sectors including Ministries of Health, Education, Justice, and Social/Human/Gender Development (Armenia, Belize, Dominican Republic, El Salvador, Iran, Kenya, Paraguay, Peru, Tanzania, Tunisia, Uganda). However, the geographic scale of these integrated government services was not clearly or consistently reported across studies. In studies that reported this information, the scale at which CCD-based or CCD-informed services were implemented—primarily in the health sector—ranged from one health facility (e.g., the Kangaroo Mother Care Program in the San Lorenzo de Los Mina Maternity and Children's Hospital; (40) [Dominican Republic]) to an entire county (e.g., routine health facility clinical services in Siaya County; (32) [Kenya]).

Some studies indicated that CCD had been embedded in national policies and strategies for ECD (Belize, Bhutan, Dominican Republic, Egypt, El Salvador, Iran, Mali, Pakistan, Peru, Uganda) but did not clarify whether or how such policies translated vertically into local-level implementation of a CCD-based or CCD-informed service. It is therefore not clear the extent to which CCD has been adopted by national governments. Additionally, only a handful of these reports provided details on how CCD had been integrated into the existing roles and responsibilities of delivery agents or the processes by which the implementation of services were supervised or monitored. This was also true for reports where CCD was delivered as an intervention outside of an existing service or system. Only two reports indicated using data from a job analysis of delivery agents to inform the integration of CCD into an existing service [(34) [Malawi]^6^; (50) [Pakistan]]. Four studies also provided data on the feasibility and impact of integrating CCD into the existing responsibilities of delivery agents ((51) [Kazakhstan, Kyrgyzstan, Tajikistan]; (25) [Mozambique]; (29) [India and Pakistan]; (50) [Pakistan]). These data show that although some delivery agents appreciated the importance of CCD and reported some improvements in their ability to coach caregivers, issues such as lack of time due to competing activities, lack of systematic practical training and supervision, and high workforce turnover rates impeded their ability to integrate CCD into their existing roles and responsibilities.

The available data on supervision indicate that supervisory contacts occurred at least once a month and consisted of different strategies such as on-the-job coaching, peer support groups, and shadowing. With respect to monitoring indicators, only five reports described the specific tool used to monitor delivery agents (*CCD Observation of Provider's Counseling Checklist* ((52) [Tanzania], (32) [Kenya], (31) [India]); *Physician Counseling Skills Scale* ((15) [Turkey]); and a locally developed *CCD Monitoring and Evaluation Surveillance System* ((46) [Belize and Anguilla]). Most remaining reports generally indicated that delivery agents had been observed administering the service, with a few (*n* = 9) indicating that a checklist had been used but did not detail how this was done or what specifically was assessed.

A larger number of reports provided data on one or more aspects of the training of delivery agents including the duration, background of trainers, learning methods used, whether refresher sessions were held, and if process and outcome evaluations of the training were conducted. Out of the 35 services that reported the duration of training, the amount of time dedicated to CCD training ranged from two days to six weeks (most services had 2–5 days of training^7^) and they were conducted by academic researchers and ECD specialists from non-governmental organizations (NGOs, e.g., UNICEF, PATH, World Vision). Twenty-one studies reported using a train-the-trainer model, where government staff, supervisors, or other individuals were trained on service administration and then subsequently trained the delivery agents. Only thirteen services reported using active learning strategies (e.g., demonstration, role plays, practice with caregivers and children) in the training process ((32) [Kenya]; (33) [Uganda]; (24) [Indonesia]; (53) [Rwanda]; (30) [Turkey]; (54) [Rwanda]; (26) [Tanzania]; (39) [South Africa]; (55) [India]; (35) [Tanzania]; (31) [India]; (36) [Egypt]; (50) [Pakistan]) and twelve reported hosting refresher sessions during service delivery ((32) [Kenya]; (33) [Uganda]; (56) [Kenya]; (25) [Mozambique]; (26) [Tanzania]; (54) [Rwanda]; (39) [South Africa]; (57) [Armenia]; (35) [Tanzania]; (58) [Kenya]; (17), (26) [Pakistan]; (59) [China]). The handful of studies (*n* = 9) that assessed delivery agents before, during, or after training generally reported improvements in knowledge about child development and competencies in delivering the service ((32) [Kenya]; (24) [Indonesia]; (30) [Turkey]; (27) [Malawi]; (55) [India]; (29) [India and Pakistan]; (36) [Egypt, Iran, Tunisia]; (31) [India]; (50) [Pakistan]).

Only nineteen reports included information about the evaluation of CCD services on children's developmental outcomes, caregivers' parenting practices, and some other child (e.g., nutrition, health) and caregiver (e.g., mental health) outcomes (see Supplementary Table S4). All but one of these reports examined short-term (i.e., immediately after exposure or within 12 months of exposure) impacts. The only long-term evaluation was conducted 2 years after exposure to a CCD-based service ((60) [Pakistan]). Both randomized (*n* = 11) and non-randomized (*n* = 8) study designs were used for these evaluations and the sample size of participants ranged from *n* = 38 to *n* = 2953. However, only nine studies used a randomized controlled trial to determine the causal impact of the service on child and caregiver outcomes. Most studies (*n* = 11 out of the 16 that assessed child outcomes) reported small-to-moderate effects of the service on children's cognitive, language, or multi-domain development, while the remaining *n* = 5 found no significant effects. Over half of the studies (*n* = 8 out of the 14 reporting caregiver outcomes) also found significant improvements in stimulation-based parenting practices and responsive caregiver-child interactions in caregivers receiving the service compared to caregivers that did not. The single long-term evaluation found continued improvements in child and caregiver outcomes two years later. Only one of the studies examining impacts on caregiving outcomes included quantitative data on fathers ((26) [Tanzania]).

### 3.2. Summary of key informant interviews

Findings from the key informant interviews are summarized below and the details of key informants (names, affiliated institution/organization, and location (country/region) of CCD experience) are presented in Panel 2.

**PANEL 2:** List of key informants^*^.

**Name**	**Affiliated institution/organization**	**Country/region of CCD experience**
Aisha Yousafzai	Harvard T. H. Chan School of Public Health	Pakistan
Amina Mwitu	Aga Khan Foundation	East Africa
Bernadette Daelmans	World Health Organization Headquarters	Global
Boniface Kakhobwe	UNICEF Headquarters	Global
Florence Kitabire	UNICEF Eastern and Southern Africa Regional Office	East and South Africa
Jane Lucas	Independent Consultant	Global
Jill Luoto	University of Southern California	Kenya
Josephine Ferla	Elizabeth Glaser Pediatric AIDS Foundation and Save the Children	Tanzania
Joyce Marangu	Aga Khan University's Institute of Human Development	East Africa
Lana Drivdal	PATH	East Africa
Maria Paula Reinbold and Patricia Núñez	UNICEF LACRO	Latin America and the Caribbean
Megan McHenry	Indiana University School of Medicine	Kenya
Melissa Gladstone	University of Liverpool	Malawi
Nafisa Shekhova	Aga Khan Development Network	Global
Paul Lynch	University of Glasgow	Malawi
Radhika Mitter	UNICEF Headquarters	Global
Tomomi Kitamura	UNICEF Middle Eastern and North African Regional Office	Middle East and North Africa
Vibha Krishnamurthy, Priyamvada Das, Namrata Edwards	Ummeed	India
Vika Sargsyan	World Vision	Armenia
Zelee Hill	University College London	India, Pakistan

^*^Any quotes included in the description of the qualitative data are anonymized. Only the type of institution/organization the informant is affiliated with is reported (academic researcher, independent consultant, or NGO staff).

The analysis grid for the first round of interviews consisted of 6 themes: (1) definition of CCD (how informants defined CCD, what they perceived its objectives to be, and what they thought was unique about CCD compared to other ECD packages), (2) justification for CCD (why informants chose to use the CCD package or not), (3) advantages and disadvantages of CCD (what informants identified as the strengths and weaknesses of CCD), (4) implementation of CCD (what informants identified as common challenges and facilitators to CCD implementation and reports of caregivers' and delivery agents' perception of CCD), (5) how to implement CCD (what advice informants would give those wishing to implementing CCD), and (6) future of CCD (informants' visions and hopes for CCD and reports of what UNICEF and the WHO can do, both in terms of leadership and additional resources, to address existing strengths and weaknesses of CCD).

#### 3.2.1. Theme 1: Definition of CCD

Many key informants defined CCD as an approach or set of recommendations intended to promote skills in delivery agents to promote child development.

“*[CCD] is created as two practical skills that all health workers should have in their contacts with young children and their families to promote and support healthy development. [These skills are] the promotion of a variety and play and communication activities and using that context to guide a responsive interaction between caregivers and their young children.”* (Academic researcher #1)

Other informants defined CCD as a training package or intervention program that promotes responsive care and stimulation for young children. Furthermore, some highlighted that the messaging of CCD goes beyond counseling as it can also be used in group sessions. Key informants described CCD's objective as to inform and train health workers and other delivery agents about child development and the need for responsive care. According to informants, elements of CCD that make it unique from other ECD packages are that it is practical and “*puts the caregiver first”* (NGO staff #1). Additionally, key informants reported that CCD shows why responsive caregiving is important for child development. Overall, informants identified CCD as being suitable for delivery agents' skills as it resembles adult learning with an emphasis on coaching and practical training.

#### 3.2.2. Theme 2: Justification for CCD

One key informant decided to use CCD after a literature review of ECD packages (Academic researcher #2). This informant stated that CCD fit well with their target beneficiaries' access to resources and that they ultimately decided to use CCD because it was already being used in the country they were working in. Some informants used CCD because it was recommended to them by a trusted ECD expert, while others were attracted to it because it is a “*WHO and UNICEF package and has this kind of seal of excellence*” (NGO staff #2). On the other hand, one informant consulted the CCD package but decided not to use it because it did not contain enough structured activities and detail. They noted that “*we were looking for a program and the CCD package was mostly bullet points”* (Academic researcher #3). This informant ended up using an ECD package with a structured curriculum that provided clearer guidance for delivery agents.

#### 3.2.3. Theme 3: Advantages and disadvantages of CCD

According to key informants, one of the main strengths of the CCD package is that it is open source and can thus be used by anyone. Informants also appreciated that the messages and trainings are flexible and can be adapted to different countries, sectors, and services. Other strengths of the package highlighted by informants included the counseling card's provision of practical advice on what caregivers can do with their children and that the overall package is suited to be administered by delivery agents in primary healthcare facilities. With respect to the weaknesses of CCD, key informants mostly identified issues with its content and implementation. These included a lack of information on the number of contacts needed to change parenting practices, lack of an explanation for why play and communication is important and why delivery agents and caregivers should invest in it, the need for more support to delivery agents after training—particularly through refresher sessions and monitoring of delivery, the lack of available and easily identifiable master trainers at the regional level, and the lack of specific messages addressing children with disabilities and caregivers' mental health.

#### 3.2.4. Theme 4: Implementation of CCD

Related to these weaknesses, informants also discussed challenges that they and their teams faced in implementing CCD. Challenges occurred at multiple levels including the caregiver/community (e.g., building trust with caregivers and other relevant community stakeholders who do not see the need or importance of CCD messages), delivery agent (e.g., high workload and turnover rates, low levels of education and lack of ECD background, lack of post-training follow-up in train-the-trainer model, difficulty in consistently using high-quality monitoring and evaluation materials, lack of specific guidance on supportive supervision), and systems (e.g., low levels of buy-in from local governments, low capacity and support from in-country and regional partners, difficulty ensuring sustainability due to lack of funding) levels. On the other hand, informants identified factors that had facilitated the implementation of CCD in their respective contexts. Common facilitators were having community champions/advocates who helped increase community engagement with CCD services, working in a centralized government system, where receiving government support at one level opened doors for working at other government levels, and working with delivery agents who saw the value and importance of CCD and were thus invested in ensuring its successful implementation.

In terms of caregivers' and delivery agents' perceptions of CCD services, most informants reported that they were positive. Caregivers in many contexts appreciated CCD because the play and communication activities helped them to better understand and interact with their children. In some contexts, caregivers who had participated in a CCD service went on to become “*champions*” (NGO staff #1) in their communities, which in some cases led fathers and other caregivers to “*[come] along to the group sessions and…participate in the home visits*” (Academic researcher #1). Overall, delivery agents also appreciated the CCD services and enjoyed delivering it. Informants indicated that delivery agents were “*happy to communicate [CCD] messages to parents*” (NGO staff #1), appreciated being able to look at children's development and wellbeing beyond medical issues, and felt that “*they can actually talk to the child and the mother as human beings as opposed to just clients”* (NGO staff #2). However, informants did clarify that although delivery agents had positive perceptions of CCD, some did not see “*counseling mums…as completely their role”* (Academic researcher #4).

#### 3.2.5. Theme 5: How to implement CCD

Implementation of CCD begins with sensitization and advocacy about why ECD is important and how the CCD package can support healthy development of the youngest children. However, while awareness about CCD has reached a number of countries around the world, key informants generally noted that advocacy efforts often did not reach the level of community (i.e., delivery agent and caregivers).

“*Advocacy doesn't trickle down to family level.”* (Academic researcher #4)

However, it was noted that when families and delivery agents experienced CCD, it was often enjoyed, which was a facilitator to implementation.

While multiple key informants noted the importance of formative research^8^ and adaptation of CCD to the local cultural context and needs (e.g., ensuring piloting, testing feasibility in both rural and urban settings), several also pointed to addressing the needs of the delivery agents (e.g., job analysis, strengthening supervision skills, and fostering discussions on why CCD and promoting ECD should be their business).

“*Supervisors need to be trained to model good teaching rather than use didactic instruction. Supervision skills are really critical.”* (NGO staff #3)

Informants also highlighted the importance of intentionality when bundling different packages:

“*It's about being thoughtful about which messages complement one another...If you're going to bundle things together, why are you bundling things together? The person delivering [the service] has to understand that…so if your training just says ‘here's another thing I want you to do' without helping [the delivery agent] navigate ‘how am I going to manage all of these in one home visit? Do they all have to be delivered in one home visit?'. If you don't do any of that homework then it's not going to work. It's just another intervention and they'll deliver the one they get paid to deliver.”* (Academic researcher #1)

#### 3.2.6. Theme 6: Future of CCD

This theme emphasized what implementers need to support quality implementation of CCD on the ground. This included suggestions ranging from expanding the training guidance to include recommendations on planning refresher trainings, creating guidance on monitoring and evaluation for CCD, and making competency standards available for assessing the skills of delivery agents. Among the most common requests was the opportunity to share experiences:

“*It would be nice to have a hub, like an online hub, where partners could post their experiences like 'look we've adapted this material and because of this, and this, and this right, and this is how we are you using it and these are the touch points where we're implementing it'. And if you have any study you put it there to show what happened, any evidence. So just to kind of have live examples of how materials are used...in systems from different parts of the world and sample materials. Right yes, so like a public learning hub would be very helpful.”* (NGO staff #2)

In some cases, key informants highlighted the need for greater clarity on content and behavior change techniques. For example, one key informant expressed that it was important to ensure that delivery of CCD was a practical experience where caregivers could try play and communication activities with their young child. While this practical approach is central to the CCD content, the response suggests the need for greater technical support and clarity for end-users. Finally, in response to emerging needs, a few key informants noted the need for a more inclusive approach to CCD expanding on how the needs of children with developmental delays and disabilities could be met and considering how to expand the global footprint to high-income settings.

## 4. Discussion

As the first systematic review of services using the CCD package, we aimed to examine how it has been implemented in different contexts and how it benefits caregivers and children. Our discussion is organized to identify how it has been integrated into government services and systems, common features of the package's content, the training of delivery agents, and engagement of beneficiaries, highlighting the strengths and limitations of each. Key informant interviews provided a deeper understanding of challenges faced when implementing the CCD package, as well as the benefits. Based on findings from the systematic review and key informant contributions, we provide some recommendations going forward.

### 4.1. Government integration

The systematic review uncovered 55 documents, of which 34 were peer-reviewed publications and 2 pre-prints. Forty-six were explicitly based on the CCD package (9). Of these, 34 had been integrated into the government health service. This was seen as a strength in that health workers with the potential for sustaining the service were trained. In most cases, it was not clear if the integration included the health system at the local level only (e.g., community health workers and clinic staff) or at the district and national levels. Most were not scaled geographically beyond the district or county level. In ten cases, CCD had been embedded in national policies and strategies for ECD, but it was not clear whether the policies had influenced implementation.

### 4.2. Content and structure of the program

For CCD-based services, the content and structure followed the CCD package, regardless of whether they were delivered in a clinic, home visit, or group session. Because the Participants' Manual used by delivery agents does not propose a structured program, implementers felt this allowed for flexibility in adapting it to their context. Some saw this as an opportunity to elaborate on the activities conducted with caregivers and so developed manuals with illustrations for delivery agents to use (e.g., SPRING Trial Team), while others used the CCD Participants' Manual and Counseling cards. Adaptations were undertaken to make materials suitable for the country, culture, and education level of the delivery agent. However, regardless of education level, most agents were initially naïve to principles of ECD and responsive play and communication. Common adaptations included translation into the local language, changes to the counseling card illustrations, and addition of local games as play activities. Another adaptation was the intensity of contacts with caregivers, varying from one 5-min session in a clinic setting to 40 fortnightly group and home visits over 2 years. Interventions that were informed by, but not based on, CCD generally deviated by considering other programs that provided more structured play and communication activities beyond what was available from the CCD package. Flexibility of the CCD package may be seen as a strength only if the curriculum developer has the expertise to insert content and structure that is needed by delivery agents and caregivers if they are unable to translate Participant Manual suggestions into practical activities.

In order to encourage caregivers to adopt the proposed practices, most services used more than one technique of behavior change. The most common was self-performance, whereby the delivery agent encouraged the caregiver to engage in the new play or communication practice with their child followed by coaching and feedback from the delivery agent. Another common technique was the use of print media, such as the counseling cards illustrating how caregivers play with their child. Because the suggested game is age-specific it may be insufficient or inapplicable two weeks later. Additional techniques that were less common included watching the delivery agent demonstrate or model a behavior, solving problems to enacting the practice, and encouraging social support from family and peers. The use of multiple techniques of behavior change has been previously associated with improvements in children's development (18).

### 4.3. Workforce

Training, supervising, and monitoring delivery agents posed a challenge. The length of training was often too short with only 2 or 3 days to cover material in the Facilitators' and Participants' Manual, whereas other trainings lasted several weeks. Most did not provide sufficient practical or clinical experience and only thirteen used any active learning strategy to train, such as, demonstration, role plays, and practice with caregivers and children. Professionals, who were better educated, did not require as much training but often found that the increase in workload was prohibitive. Paraprofessionals and volunteers required more training but complained about the lack of refresher courses and face-to-face supervision. Some had monthly supervision with on-the-job coaching (e.g., (50)), and nine reported using a checklist to observe and provide feedback. Only a few studies reported using an assessment of delivery quality or knowledge after training [e.g., (30)]. Several implementations developed their own supervisory content and schedule; a few implemented refresher trainings at regular intervals [e.g., (57, 58)].

### 4.4. Beneficiaries

The intended beneficiaries in all cases were the child, particularly those under 3 years, and their caregivers, mainly mothers. Although CCD was implemented in urban and rural settings, the majority targeted disadvantaged families in rural areas. Recent implementations have been directed at vulnerable groups such as caregivers of children born prematurely, with visual impairments, and with other physical and/or cognitive disabilities. These interventions have required considerable adaptation to accommodate the needs of these children and their caregivers. Some have also addressed nutritional problems of stunted children and the well-being of mothers at risk of depression.

Only nineteen reports described their evaluation of beneficiary outcomes for children and/or caregivers. Approximately half were CCD-based and half CCD-informed. Both randomized and non-randomized designs were used. This is a clear limitation. Of the 16 that assessed child outcomes, eleven reported small-to-medium effects of the service on children's cognitive, language, or multi-domain development, while the remaining five found no significant effects. Of the 14 assessing caregiver outcomes, eight found small-to-medium improvements in stimulation-based parenting practices and/or responsive caregiver-child interactions in caregivers receiving the service compared to caregivers who did not. These are promising findings that call for more interventions to be evaluated using convincing designs and measures. Evidence that an intervention is effective, feasible, and acceptable is important before scaling it up.

### 4.5. Recommendations

As noted previously, reports did not often elaborate on how the CCD program was being implemented at scale, both within the government system and across the country. This limitation has been addressed in Belize and could be a solution for others. To support the monitoring and evaluation of CCD services at scale, Belize has developed a CCD monitoring and evaluation surveillance system [CCD-MESS (46)]. CCD-MESS was developed in 2019 to set common service provision standards for all service providers involved in CCD delivery. The system defines the building blocks of a CCD session, the schedule of sessions depending on the child's condition (e.g., premature, stunted, disability, or no special condition), and establishes that all delivery agents should be trained at least once a year. Delivery agents are required to submit monthly reports to help track the 12 indicators highlighted in the CCD-MESS. The system also defines a checklist for use by supervisors when monitoring delivery agents (46). The development of similar monitoring and evaluation systems can be used to track the quality and effectiveness of large-scale early childhood services.

Additional challenges in implementing at scale were raised by key informants who were also asked to recommend solutions. Challenges included lack of buy-in from local and national levels of government, low capacity and support from in-country and regional partners, and difficulty ensuring sustainability due to lack of funding. Key informants also highlighted challenges to CCD implementation at the caregiver- (e.g., building trust with caregivers and other relevant community stakeholders who may not see importance of CCD messages) and delivery agent- (e.g., high workload and turnover rates, lack of post-training follow-up training, difficulty in consistently using high-quality monitoring and evaluation materials) levels. When asked about potential solutions to these challenges, key informants suggested expanding training guidance to include recommendations on planning refresher trainings, creating technical guidance on monitoring and evaluation for CCD, and developing competency standards for assessing the skills of delivery agents. Executing these and other solutions will require communication and coordination between UNICEF and WHO (both the headquarters and regional offices) and early childhood researchers and program implementers to ensure sustainable implementation and effective impact of the CCD package on child and family outcomes.

## 5. Conclusion

Overall, CCD has helped raised awareness of strategies to promote ECD among key program implementers in low- and middle-income countries, especially in the health and nutrition sectors. The open access to the package and the flexibility that permits contextualization and adaptation to culture, context and delivery system is a strength. However, access to technical support is important when adapting and rolling out to ensure the core recommendations are appropriately implemented. This review provides a timely summary of how CCD has been implemented in various contexts, highlighting key strengths that can be built on and challenges pertaining to implementation roll-out for scale and sustainable uptake that need to be addressed. While many of the implementation challenges are not unique to CCD and have been noted in the broader literature that points to the lack of scale-up of effective parenting interventions, here is a window of opportunity to reflect on these results and consider how the CCD package may be expanded to support roll-out in systems for scale (e.g., guidance on how to introduce and foster buy-in for CCD to policy makers, building skills for trainers and supervisors, monitoring CCD in health and nutrition systems) and platforms created to share lessons from large scale implementation efforts. Given new realities of the youngest global citizens and their caregivers, including recovering from the consequences (psychologically and economically) of the COVID-19 pandemic and facing the growing risks of conflict, climate change, and increasing inequities in society, multiple parenting packages and innovations will be needed in which CCD can play a key role in offering solutions.

## Data availability statement

The original contributions presented in the study are included in the article/Supplementary material, further inquiries can be directed to the corresponding author.

## Author contributions

MA and AY developed the study concept. MA, FA, and CW conducted the literature review and data extraction. MA prepared the first draft of the manuscript. All authors provided critical feedback on drafts of the manuscript and were involved in the analysis of data from the key informant interviews. All authors contributed to the article and approved the submitted version.

## Footnotes

^1^That is, noticing the child's signals and providing timely and developmentally appropriate responses (14).

^2^Counseling cards are a job/visual aid for delivery agents with a key play and communication activity shown for each of the following age groups: newborn/birth up to 1 week, 1 week up to 6 months, 6 months up to 9 months, 9 months up to 12 months, 12 months up to 2 years, and 2 years and older.

^3^The checklist for counseling on care for child development guides delivery agents to identify practices to support the child's development, counsel the caregiver (look, ask, listen), and praise and advise caregivers.

^4^Facilitator Notes is a training manual designed for 3.5 days of training. The notes are to be used by the trainers to promote skills for guiding caregivers to stimulate their young children and interact responsively while playing and communicating with their young children.

^5^Note that the play and communication guide developed by Yousafzai et al in Pakistan was used by Jeong et al in Tanzania and by the SPRING Trial team in India and Pakistan.

^6^Gladstone et al. used the job analysis of health surveillance assistants in Malawi from Phuka et al. (49) to inform the integration of CCD into health services.

^7^Note that the recommended duration of training—according to the Facilitator Notes—is 3.5 days.

^8^Data from the systematic review show that 17 reports indicated that formative research had been conducted prior to the implementation of the described CCD-based or CCD-informed service (Supplementary Table S1).
