# Supplementary material for: Implementation of UNICEF and WHO's care for child development package: Lessons from a global review and key informant interviews
Source: Front Public Health. 2023 Feb 16;11:1140843. doi: 10.3389/fpubh.2023.1140843 (PMC9978394; doi:10.3389/fpubh.2023.1140843)
Supplement: Supplementary file 1 [file Table_1.docx]

**APPENDIX 1 – KEY INFORMANT INTERVIEW TOPIC GUIDES AND ANALYSIS GRID**

**Topic guide for informants who consulted, implemented, or evaluated CCD**

**[Section 1: Introduction]**

*Hello, my name is <name of interviewer> and I am working on a desk review of UNICEF and the WHO’s Care for Child Development package. Thank you so much for taking the time to speak with me today. I would like to speak with you for about an hour about your work with Care for Child Development OR the parenting programme described in <insert country name and title and year of research article/report if relevant>. I would like to know in detail about various aspects of this project with regards to its implementation and evaluation. Do you have any questions for me before we begin?*

**[Section 2: General questions about involvement in CCD implementation and evaluation – all informants]**

1. Can you please tell me about your role in the organization/institution you work in and in the implementation and/or evaluation of the parenting programme?
   1. ***This probe should only be made to an informant who works in a non-governmental organization:*** What countries does your organization/institution do ECD work in?
2. ***This question should only be asked to informants who worked on a programme for which data have not been extracted***: Can you describe the implementation strategy used to deliver your parenting programme?
   1. *Probes*: Was it a new programme or integrated into an existing service? Who was the target group? Who were the delivery agents? What setting (home/clinic/multiple) was the programme delivered in and what delivery modality (individual/group/both) was used? How long did sessions last and how frequently were they held? What was the supervision strategy (how often, who, any tools used)? How did you monitor fidelity and delivery?
   2. *Probe*: How many contacts do you expect are needed with parents/caregivers to ensure that they and their child are benefitting from the programme (i.e., that parent practices have changed and that child development has improved)?
3. Can you please describe the training strategy for delivery agents?
   1. *Probes*: How many people were in a single training? Who was the trainer? Did training include an active learning component (i.e., opportunities for delivery agents to practice what they were learning with each other or with caregivers and children)? How many days (out of total training days) were dedicated to active learning? Was there an evaluation of delivery agents’ competence/knowledge at any point (before, during, after) throughout the training process? Was there an evaluation of the quality of the training by trainees, the trainer, or an independent person?

***I noticed that there were some components of Care for Child Development in the programme and wanted to know more about your experiences with it.***

1. Can you please tell me about the process through which your team decided on which parenting package to use and why you chose (or did not chose) Care for Child Development?
   1. *Probe*: Do you find that CCD is a system-level framework that is compatible with specific services/programs/interventions that are found to be effective?
   2. How would you describe Care for Child Development to someone who had never heard about it before?
   3. What is unique about Care for Child Development compared to other ECD or parenting packages?
2. What Care for Child Development materials and/or training activities did you use in your program?
3. *Probe*: For example, manuals, job aids, training instructions, messages, monitoring and evaluation framework
4. What was the experience with and knowledge of Care for Child Development of the individual(s) who trained delivery agents?
5. What adaptations were made to the content of Care for Child Development in your context? This can include translations of the material, addition or removal of specific topics/themes, and changes to the manual and/or job aids.
   1. *Probe*: Why did you make these changes?
6. What adaptations were made to the training of delivery agents indicated in the Care for Child Development Manual?
   1. *Probe*: Why did you make these changes?
7. ***This question should only be asked to informants who worked with delivery agents who are part of a government service to implement CCD***: To what extent was Care for Child Development integrated into the daily work of the delivery agents?
8. Was Care for Child Development integrated into their routine contacts with caregivers/children or added as a separate contact?
9. How did implementing Care for Child Development impact the delivery agents’ existing roles and responsibilities?
10. Are delivery agents still implementing Care for Child Development?
11. ***This question should only be asked to informants who worked with delivery agents who are part of a government service to implement CCD***: What technical support did you (or your implementing partner) provide to the government service in question to help train and supervise delivery agents as they implemented Care for Child Development?
12. *Probe*: Was/is there capacity within the system to lead training and supervision?
13. What tools (if any) were used to monitor implementation of the intervention (for example, contacts with caregivers/children, intervention delivery observed, supervisory contacts, delivery agents’ and caregivers’ acceptance of intervention)?
    1. *Probe*: What monitoring and evaluation framework (if any) was used?
    2. *Probe*: If programme was integrated into government service: are any of these tools used by the government service?
14. Have you evaluated the impact of CCD on caregivers or children? If so, can you please share any reports with me?
    1. *Probe*: What tools were used to assess child development? Caregiver outcomes? What study design was used for the evaluation?

**[Section 3: Barriers and facilitators to CCD implementation – all informants]**

1. What barriers/challenges did you face in the implementation and delivery of Care for Child Development in your context?
2. *Probe*: At the caregiver/child level; at the delivery agent level; at the systems level (i.e., government programme, implementing partner, other stakeholders such as community members)
3. Which factors facilitated the implementation and delivery of Care for Child Development in your context?
4. *Probe*: At the caregiver/child level; at the delivery agent level; at the systems level (i.e., government programme, implementing partner, other stakeholders such as community members)

**[Section 4: Suggestions/recommendations for future implementation of CCD – all informants]**

1. What are your suggestions or recommendations for those who wish to implement Care for Child Development in the future?
   1. *Probe*: With respect to the written package, DVD, training, curriculum, monitoring and evaluation framework
2. What can UNICEF and the WHO do to better support future implementations of Care for Child Development?
3. ***This question should only be asked to an informant who works in a non-governmental organization:*** Does your organization/institution have a global or regional hubs to facilitate communication between regional groups on CCD implementation?

**[Section 5: Specific questions about CCD implementation and evaluation – some informants]**

*The interviewer will write down specific questions based on discussion so far to ask informants.*

**[Section 6: Conclusion]**

*Those were all my questions about Care for Child Development. Is there anything else about Care for Child Development that you would like to share?*

*Thank you so much for taking the time to speak with me. Do you have any questions for me? Thank you again for your time. I will share the final report with you once it is published.*

**Analysis grid for first round of key informant interviews**

| **Theme** | **Specific questions** |
| --- | --- |
| *Definition of CCD* | How do informants define CCD? What does it consist of? |
|  | What is the objective of CCD? |
|  | What is unique about CCD? |
| *Justification for CCD* | Why do informants and their teams use CCD? |
|  | Why do some choose to use other packages? |
| *Advantages and disadvantages of CCD* | What are the strengths of CCD |
|  | What are the weaknesses of CCD? |
| *Implementation of CCD* | What are some common challenges informants and their teams have faced in implementing CCD? |
|  | What are some common facilitators or successes informants and their teams have experienced in implementing CCD? |
|  | What are caregivers and delivery agents’ perception of CCD? |
| *How to implement CCD* | What are some common pieces of advice informants have for those wishing to implement CCD? |
| *Future of CCD* | What can UNICEF and the WHO do to address existing strengths and weaknesses of CCD? |
|  | What additional resources are needed to better support CCD implementation and evaluation? |
|  | What are informants’ visions/hopes for CCD? |

**Topic guide for informants involved in development or management of CCD**

*Hello, my name is <name of interviewer> and I am working on a desk review of UNICEF and the WHO’s Care for Child Development package. Thank you so much for taking the time to speak with me today. I would like to speak with you for about an hour about your role in the development and administration of training workshops for the Care for Child Development Package. Do you have any questions for me before we begin?*

1. Can you please tell me about your role with UNICEF/WHO, including your involvement in the Care for Child Development Package?
2. How do you define Care for Child Development, what is it and what is its objective?
3. What is the leadership role you envision for UNICEF and the WHO in the management of the Care for Child Development package? For example, in promoting and supporting the implementation of Care for Child Development.

Questions for UNICEF/WHO staff

1. What is your vision for the Care for Child Development centres of excellence that you want to invest in (e.g., the Aga Khan University’s Institute of Human Development)?
   1. Is there a role for regional centres of excellence in supporting Care for Child Development?
2. How does Care for Child Development align with the Nurturing Care Framework?
3. How does Care for Child Development align with the Care for Caregiver and Thinking Healthy packages?
4. How does Care for Child Development fit in with the ECDAN parenting initiative?
5. What kind of evidence is needed to strengthen the reputation and use of the Care for Child Development package in the field of parenting interventions?
   1. What evidence does Care for Child Development contribute to the 2018 WHO systematic review and recommendations on parenting interventions?
6. What monitoring indicators are needed to track the progress of the Care for Child Development package?
7. What are your thoughts on the recommendations from the draft report?

Questions for CCD Master Trainer

1. How well are the individuals you train (e.g., providers at the health facility level) able to train and sustain community health workers/volunteers?
   1. How do you define a master trainer? What competencies do they need to have to obtain this designation? How are these competencies evaluated?
2. What kinds of questions do countries/organizations reach out to you with after you have trained them?
   1. What follow-up support do you provide to countries/organizations who reach out to you after you have trained them?
   2. What technical (or other) supports do countries/organizations need beyond the training that you provide?
3. What is your vision for expanding training capacity for Care for Child Development?
   1. What technical tools (additional or existing) are needed to facilitate this expansion?
4. If there was an opportunity to update other Care for Child Development materials, what would that look like for you?

**APPENDIX 2 – TABLES (S1-S4) AND REFERENCE LIST OF ALL STUDIES INCLUDED IN REVIEW**

**Table S1.** Content and structure of interventions and services using the Care for Child Development Package

| **Author, year, country (*implementing partner*)** | **Peer-reviewed?** | **Delivery modality** | **Intensity^1^** | **CCD-based or CCD-informed** | **Formative research?** | **Adaptations^2^ to CCD?** | **CCD bundled with another package?** | **NCF components included** | **Structured session guide/manual** | **Use of job aids** | **Behaviour change techniques^3^** |
| --- | --- | --- | --- | --- | --- | --- | --- | --- | --- | --- | --- |
| Ertem et al., 2006, Turkey* | Yes | Clinic visits | 2 weekly sessions | CCD-based | Yes |  |  | Opportunities for early learning | Yes | Yes | Performance [other, self] |
| Jin et al., 2007, China* | Yes | Clinic visits | 2 biannual 30-60 min sessions | CCD-based | Yes | Yes, translation to Chinese |  | Good health,  Opportunities for early learning | Yes | No | Media [print], performance [other, self], problem-solving |
| Ertem et al., 2009, Turkey – ***training evaluation*** | Yes | Clinic visits |  | CCD-informed | No | Yes, expansion to include concepts related to mental health and emotional and cognitive development |  | Opportunities for early learning | No | No |  |
| Engle et al., 2011, Kazakhstan, Kyrgyzstan, and Tajikistan* | No | Clinic visits | Different services:  -One 5-min session | CCD-based | Yes |  | Yes | Adequate nutrition, Good health, Opportunities for early learning, Responsive caregiving | No | Yes (Kazakhstan) |  |
| Jones, 2012, Mozambique* (*Aga Khan Foundation*) | No | Home visits | 3 sessions | CCD-based | No |  |  | Opportunities for early learning | Yes | No | Media [print]; Performance [self] |
| †Lingam et al., 2014 and SPRING Trial Team personal communication, India and Pakistan* | Lingam (yes)  Personal communication (no) | Home visits | 27 monthly 10-45-minute home visits over 2 years | CCD-based | Yes | Yes, translation into local languages, adaptation to local contexts, and expansion of play and communication activities | Yes; WHO complementary feeding guidelines | Adequate nutrition, Opportunities for early learning, Responsive caregiving, | Yes | Yes | Media [print], Performance [self, other], Problem-solving, Social support [family] |
| †Thorne 2014, Kenya, Tanzania*, Uganda (*Aga Khan Foundation*) – ***training report*** | No | Clinic visits, group sessions, and home visits | Different services: Monthly sessions | CCD-based | No | Yes, translation to Kiswahili and Luganda. Also, changes to mode of delivery and level of reading to suit delivery agents’ backgrounds |  | Opportunities for early learning, Responsive caregiving | Yes | Yes |  |
| Yousafzai et al., 2014, 2015, 2018 Pakistan* | Yes | Group sessions and home visits | 20 80-min monthly group sessions and 20 11-30-min monthly home visits over 21.5-24 months | CCD-based | Yes | Yes, changed child age groups of counselling cards, added resource kit w/ sample learning materials and picture book, and expanded play and communication activities | Yes, nutrition education package | Adequate nutrition, Opportunities for early learning, Responsive caregiving, Safety and security | Yes | Yes | Materials, performance [self], problem-solving |
| Holding, 2015, Mali* (*UNICEF, Handicap International, BØRNEfonden*) | No | Group sessions and home visits |  | CCD-based | Yes | Yes, contextualization of materials and translation to French |  | Opportunities for early learning, Responsive caregiving, Good health | No | No | Media [print] |
| PATH, 2015, Mozambique* (*PATH*) | Yes | Playbox sessions | A few days per week | CCD-based | Yes | Yes |  | Opportunities for early learning, Responsive caregiving | No | Yes | Media [print] |
| Rockers et al., 2016, Zambia | Yes | Group sessions and home visits | 20 group sessions and 23 home visits held fortnightly over 12 months | CCD-informed | No |  |  | Adequate nutrition, Good health, Opportunities for Early Learning | Yes | No | Performance [self], social support [community] |
| Bayitondere et al., 2018, Rwanda* | Yes | Clinic visits and group sessions |  | CCD-based | No |  | Yes, Pediatric Development Clinic model | Opportunities for early learning; responsive caregiving | No | No |  |
| Fisher et al., 2018, Vietnam* – ***study protocol*** | Yes | Group sessions and home visits | 19 60-90-min group sessions every 2-4 weeks over 18 months. One 45-60-min home visit | CCD-based | Yes | Yes, translation to Vietnamese and cultural adaptations | Yes, many^4^ | Adequate nutrition, Good health, Opportunities for Early Learning, Responsive caregiving, Security and safety | Yes | Yes |  |
| †Gladstone et al., 2018, Malawi* | Yes | Group sessions and home visits | 12 45-min group sessions and 12 30-min held fortnightly over 6 months | CCD-based | Yes |  | Yes, IMCI nutrition, WASH, and health content | Adequate nutrition, Good health, Responsive caregiving | Yes | Yes | Materials, Media [AV, print], Social Support [family and community], performance [self, other], problem-solving |
| †Lynch et al., 2018 and Gladstone et al., 2017, Malawi* | Yes | Home visits | 12 60-min session held fortnightly over 6 months | CCD-based | Yes | Yes, adapted for children w/ visual impairments and expansion of play and communication activities |  | Opportunities for early learning; responsive caregiving | Yes | Yes | Materials, Media [print], performance [other, self] |
| †Rosales et al., 2019 and World Vision International 2017, Armenia (*World Vision*) | Yes (Rosales);  No (World Vision International) | Clinic visits, group sessions, and home visits for those who missed group | 8 90-120-min bi-monthly group sessions | CCD-informed | Yes | Yes, adapted to local parenting practices | Yes, World Vision Armenia's maternal, newborn, and child health programme | Adequate nutrition, Good health, Opportunities for early learning, Responsive caregiving, Safety and security | Yes | Yes | Performance [self], Materials, Social Support [Family] |
| UNICEF 2019, Paraguay* (*UNICEF*) | No | Group sessions and home visits | Different services: weekly home visits, monthly home visits and biweekly group sessions, weekly individual and group sessions | CCD-based | No | Yes, translation to Spanish and Guarani |  | Adequate nutrition, Opportunities for early learning, Responsive caregiving | Yes | Yes | Performance [other, self], Social support [family] |
| Zhou et al., 2019, China* | Yes | Clinic visits and group sessions | 3+ available per month over 2 years | CCD-based | No |  |  | Adequate nutrition, Good Health, Responsive caregiving, Opportunities for early learning, Safety and Security | No | No | Materials, Performance [self], Problem solving |
| Barnhart et al., 2020, Rwanda | Yes | Home visits | 12 60-90-min weekly sessions over 3-4 months | CCD-informed | Yes | Yes, translated to Kinyarwanda | Yes, Family Strengthening Intervention for HIV | Adequate nutrition, Good Health,  Opportunities for early learning,  Responsive caregiving, Safety and Security | Yes | No | Performance [other, self], Problem solving, Social support [family] |
| Betancourt et al., 2020, Rwanda | Yes | Home visits | 15-22 sessions over 4-8 months | CCD-informed | Yes | Yes, translated to Kinyarwanda | Yes, Family Strengthening Intervention for HIV | Adequate nutrition, Good Health,  Opportunities for early learning,  Responsive caregiving, Safety and Security | Yes | Yes | Performance [other, self], materials, problem-solving |
| Rao et al., 2020 and Chan et al., 2021, Bhutan* (*UNICEF*) | No (Rao); Yes (Chan) | Group sessions at a clinic |  | CCD-based | No |  |  | Adequate nutrition, Good Health,  Opportunities for early learning,  Responsive caregiving, Safety and Security | No | No |  |
| Shah et al., 2020, India | Yes | Clinic visits | 1 10-min session | CCD-informed | No | Yes, translated to Kannada, Hindi, and Marathi |  | Opportunities for early learning, Responsive caregiving | No | No | Performance [other, self], Materials |
| Shi et al., 2020, China | Yes | Clinic visits, telephone support | 2 120-min sessions, 4 months apart | CCD-informed | No | Yes, translated to simple Chinese |  | Adequate nutrition, Opportunities for early learning | No | No | Media [print] |
| Xu et al., 2020, China – ***study protocol*** | Yes | Group sessions | 42 45-min fortnightly (6-23m) and monthly (24-30m) over 24 months | CCD-informed | Yes |  | Yes | Adequate nutrition, Good health, Opportunities for Early Learning, Responsive caregiving, Security and safety | Yes | No |  |
| †African Population Health Research Centre, 2021, Kenya* (*PATH*) | No | Clinic visits, home visits, Playbox sessions | 5 to 15-minute routine clinic visits (birth, 6 weeks, 10 weeks, 14 weeks, 6 months, 9 months, etc.). Monthly home visits | CCD-based | No | Yes, translation to Kiswahili. Adaptation of CCD content and illustrations. | Yes, IMCI | Adequate nutrition, Good health, Opportunities for Early Learning, Responsive caregiving, | Yes | Yes | Materials, Performance [other] |
| †Aga Khan University and Aga Khan Foundation, 2021, Kenya* (*Madrasa Early Childhood Programme*) | No | Clinic visits, group sessions, home visits | Regular clinic and home visits. Monthly group sessions. | CCD-based | No | Yes, translation to Kiswahili. Addition of community dialogue sessions and information, education, and communication materials for improving awareness of ECD. |  | Good health, Opportunities for early learning, Responsive caregiving | No | Yes | Materials, Performance [self] |
| Aga Khan University and Aga Khan Foundation, 2021, Syria (*AKDN*) | No | Clinic visits | 15 clinic visits over 5 years (7 in 1^st^ year and 8 from ages 1-5) | CCD-informed | No | Yes, translation to Arabic. Incorporation of CDC materials on ECD and adaptation to local context. |  | Good health, Opportunities for early learning, Responsive caregiving | No | Yes | Materials |
| †Aga Khan University and Aga Khan Foundation, 2021, Tanzania* (*Madrasa Early Childhood Programme and Elizabeth Glaser Pediatric AIDS Foundation*) | No | Clinic visits, group sessions, home visits, playbox sessions |  | CCD-based | No | Yes, translation to Kiswahili. Adaptations included contextualization of illustrations and recommendations, addition of security & safety and disability content, stronger focus on responsive caregiving, addition of more play and communication activities, introduction of group counselling, addition of video materials, and revision of M&E tools. |  | Adequate nutrition, Good health, Opportunities for early learning, Responsive caregiving, Safety and security | No | Yes | Materials, Media [AV], Performance [self], |
| †Aga Khan University and Aga Khan Foundation, 2021, Uganda* (*Madrasa Early Childhood Programme and Save the Children*) | No | Clinic visits, group sessions, home visits | Monthly home visits | CCD-based | No | Yes, translation to Luganda and Kakwa. Adaptations included expanding content to 3-year-olds and tailoring content to fathers, teenage parents, HIV+ parents, and parents struggling with alcoholism | Yes, IMCI | Adequate nutrition, Good health, Opportunities for early learning, Responsive caregiving | No | Yes | Materials |
| Akhmadi et al., 2021, Indonesia* – ***training evaluation*** | Yes |  |  | CCD-based | No | Yes, addition of traditional games, Javanese music, and local forms of advice-giving and expansion of play and communication activities |  | N/A | No | Yes |  |
| Dovel et al., 2021, Malawi* (*Partners in Hope*) | Yes | Group sessions | 8-24 sessions ~1-3 months apart over 24 months | CCD-based | Yes | Yes, also taught to identify and respond to age-appropriate developmental milestones |  | Good health, Opportunities for early learning, Responsive caregiving | No | No | Social Support [community] |
| Jensen et al., 2021, Rwanda | Yes | Home visits | 12 60-min weekly sessions over 3 months | CCD-informed | Yes |  | Yes, Family Strengthening Intervention for HIV | Adequate nutrition, Good health, Opportunities for Early Learning, Responsive caregiving, Security and safety | Yes | No | Performance [self], Social support [community and family], problem-solving |
| Jeong et al., 2021, and Bliznashka et al., 2022 Mozambique* (*PATH*) – ***qualitative evaluation*** | Yes | Clinic visits, group sessions, home visits | Different services: regular clinic visits (sick and well child consultations), regular home visits, regular group sessions | CCD-based | Yes | Yes, replacement of text with more visual representations and inclusion of local play activities, expansion of play and communication activities |  | Adequate nutrition, Good health, Opportunities for early learning, Responsive caregiving | No | Yes | Media [print], Performance [other], Social support [family] |
| UNICEF 2021, Belize and Anguilla* (*UNICEF and PAHO/WHO*) | No | Group sessions, home visits | Different services  -National MCH and CHW programmes – monthly home visits and community group sessions.  -Roving caregivers programme – 1-hour weekly home visits. | CCD-based | No | Yes, expansion to services beyond health sector, engagement of fathers and other family members, inclusion of guidance and content on violence prevention and caring for children with developmental delays and/or disabilities. Additional adaptations were made to training manual and duration. | Yes; violence prevention technical seminar | Good health, Opportunities for Early Learning, Responsive caregiving, Safety and security | Yes | Yes | Materials, Performance [self] |
| UNICEF 2021, the Dominican Republic* (*UNICEF and PAHO/WHO*) | No | Clinic visits, home visits | Different services: Kangaroo Mother Care - ~weekly clinic visits | CCD-based | No | Yes, same as in Belize |  | Good health, Opportunities for Early Learning, Responsive caregiving, | Yes | Yes | Social support [family] |
| UNICEF 2021, El Salvador* (*UNICEF and PAHO/WHO*) | No | Clinic visits, group sessions, home visits | Different services:  -Family circles – 2-hour twice weekly group sessions | CCD-based | No | Yes, same as in Belize |  | Opportunities for Early Learning, Responsive caregiving, Security and safety | Yes | Yes | Performance [self], Social support [family] |
| UNICEF 2021, Peru* (*UNICEF and PAHO/WHO*) | No | Clinic visits, group sessions, home visits | Different services:  -Growth and Development Monitoring (CRED) – ~45-minute regular check-ups till child’s 5^th^ birthday | CCD-based | No | Yes, same as in Belize. Additionally, contextual modifications were made to training package and visual design of counselling cards. |  | Adequate nutrition, Good health, Opportunities for Early Learning, Responsive caregiving | Yes | Yes | Media [print], Performance [self] |
| †UNICEF 2021, Tunisia, Iran, Egypt* (*UNICEF*) | No | Clinic visits, group sessions | Varied | CCD-based | No | Yes, translation into Arabic and Farsi. Adaptations to local contexts. Additional content on positive parenting and fathers. | Yes, IMCI (Egypt) | Adequate nutrition, Good health, Opportunities for Early Learning, Responsive caregiving,  Safety and security | No | Yes | Media [print], Materials, Performance [self] |
| †Antelman et al., 2022, Tanzania (*Elizabeth Glaser Pediatric AIDS Foundation*) | No (***pre-print***) | Clinic group sessions, home visits, radio programming | Monthly home and clinic visits, daily radio programming | CCD-informed | No | Yes, adaptation for group format and addition of play and communication activities |  | Good health, Opportunities for Early Learning, Responsive caregiving | No | Yes | Materials, Media [audio, AV, print], Performance [self, other] |
| Bahari Gharehgoz et al., 2022, Iran* | Yes | Group sessions | 4 2-hour weekly sessions | CCD-based | No |  |  | Opportunities for Early Learning, Responsive Caregiving |  |  | Performance [self], Problem-solving |
| Bemanalizadeh et al., 2022, Iran* | No (***pre-print***) | Group sessions | 5 ~45-min sessions in 3^rd^ trimester and child’s age 2-6 weeks, 2-6 months, 6-9 months, and 9-12 months | CCD-based | No |  |  | Adequate nutrition, Good health, Opportunities for early learning, Responsive caregiving, Safety and security | No | Yes | Materials, Media [AV], Performance [self] |
| †Ummeed 2022, India (*Ummeed*) | No | Group sessions and home visits | At least once a month | CCD-informed | No | Yes, translation into Hindi and Gujarati. Inclusion of content for children with disabilities and caregiver mental health. |  | Opportunities for early learning, Responsive caregiving | No | Yes | Media [AV], Performance [self] |
| Jeong et al., unpublished manuscript, Tanzania* (*Global Communities*) | Yes (under review) | Group sessions and home visits | 120-minute group sessions held biweekly over 9 months followed by biweekly home visits over 3 months due to COVID-19 | CCD-based | Yes | Yes, adaptation to group format, engagement of fathers, addition of content on positive discipline, and expansion of play and communication activities | Yes; enhanced nutrition package^5^ | Adequate nutrition, Opportunities for early learning, Responsive caregiving, Safety and security | Yes | Yes | Performance [other, self], Problem-solving, Social support [community and family] |
| †McHenry et al., unpublished manuscript, Kenya* | Yes (under review) | Group sessions | 10 90-minute sessions held fortnightly over 6 months | CCD-based | No | Yes, adaptation to group formant, expansion of play and communication activities, and inclusion of content on financial planning |  | Adequate nutrition, Opportunities for early learning, Responsive caregiving, Safety and security | Yes | Yes | Performance [self], Problem-solving, Social support [community] |

Note: Missing information (i.e., empty cells) indicates that data were not clearly provided

†Extracted data were supplemented by information provided in key informant interview(s)

*CCD-based service

AV = audio visual. CHW = community health worker. IMCI = integrated management of childhood illnesses. M&E = monitoring and evaluation. MCH = maternal and child health. NCF = Nurturing Care Framework. PAHO = Pan American Health Organization. WHO = World Health Organization.

^1^Intensity refers to the duration of the intervention/service and the frequency and length of sessions

^2^Adaptations refer to 1) translations of CCD materials, 2) adaptations to counselling cards and other materials to make them more suitable for the local context (e.g., using locally drawn images, inclusion of local play activities), and 3) modifications to the content of CCD materials (e.g., expansion of play and communication activities, addition of new themes, creation of a structured curriculum)

^3^Behaviour change techniques refer to the strategies used by the intervention/service to facilitate behaviour change in caregivers: materials = provision of materials such as books or play objects; media [AV] = use of media such as radio/audio or TV/video to convey messages; media [print] = use of print media such as pamphlets, posters, flipcharts to convey messages; performance [other] = use of demonstrations by delivery agents or another caregiver to model a behaviour]; performance [self] = caregiver themselves practicing with their child and getting feedback or coaching; problem-solving = identification of barriers and facilitators to behaviour change and solutions to overcoming barriers; social support [family] = leveraging intervention recipient’s relationship with family members as a source of support to facilitate behaviour change; social support [community] = leveraging intervention recipient’s relationship with community members and resources as a source of support to facilitate behaviour change (Briscoe & Aboud 2012, *Social Science & Medicine*; Aboud & Yousafzai 2015, *Annual Review of Psychology*)

^4^Fisher et al., 2018 – CCD was bundled with other packages including Sisters for Life component of the IMAGE trial, the What Were We Thinking Programme, and the Thinking Healthy Programme

^5^Jeong et al., unpublished manuscript – CCD was bundled with an enhanced nutrition package which included content on infant and young child feeding, dietary diversity, water, sanitation, and hygiene practices, food access, psychosocial wellbeing, gender equity, intra-household resource allocation, partner communication, and household decision-making

**Table S2.** Setting and beneficiaries of interventions and services using the Care for Child Development Package

| **Author, year, country (*implementing partner*)** | **Setting** | **Beneficiaries** | **Beneficiaries’ participation** | **Beneficiaries’ programme acceptance** | **Secondary beneficiaries** |
| --- | --- | --- | --- | --- | --- |
| Ertem et al., 2006, Turkey* | Urban, Ankara | Caregivers of children ≤24 months upon visiting a clinic that serves low- and middle-income families. Families were excluded if they lived outside of Ankara or were moderately-severely ill as judged by a screening physician | N=259 caregiver-child dyads were evaluated | Majority of caregivers reported remembering the intervention and messages, but not all of them were shown to act on these messages. Caregivers reported being satisfied with delivery agent’s services. |  |
| Jin et al., 2007, China* | Rural, Chang Feng county, An Hui province | Parents of children aged 0-2 years | N=100 caregiver-child dyads were evaluated | Post-intervention questionnaire: mothers indicated that proposed activities were feasible |  |
| Ertem et al., 2009, Turkey – ***training evaluation*** | Urban, Istanbul, Izmir, Mersin, and 2 unnamed cities | *Training of general practitioners and nurse-midwives* | *All agreed to participate in study* | *Questionnaires and focus group discussions: delivery agents identified the strong points of the programme as the content and materials of the training programme, the ease of applicability of the skills gained, the expanded CCD messages, and their improved ability to engage caregivers. Key programmatic issues included focus on improved competence and knowledge rather than comprehensive programme that changed practices and incorporated child development into health care delivery and lack of ongoing feedback for delivery agents after implementation* |  |
| Engle et al., 2011, Kazakhstan, Kyrgyzstan, and Tajikistan* | Country-wide | Mothers of young children | N=118 (Tajikistan) and n=112 (Kyrgyzstan) caregiver-child dyads were evaluated | Exit poll: mothers reported being satisfied with clinic visit |  |
| Jones, 2012, Mozambique* (*Aga Khan Foundation*) | Rural, Cabo Delgado province | Mothers of infants ≤ 2 years old | N=500 caregiver-child dyads were exposed to intervention/service. In-depth interviews were conducted with 8 mothers: 3 had received one counselling visit, 2 received 2 visits, and 3 received 3 visits | In-depth interviews (n=8): most mothers enjoyed programme and wanted it to continue, but there was some confusion about programme’s purpose (e.g., some mothers thought the purpose of teaching the child how to play with toys was to enable the mother to leave the child alone rather than encouraging interaction and responsiveness) |  |
| †Lingam et al., 2014 and SPRING Trial Team personal communication, India and Pakistan* | Rewari district (India) and Rawalpindi district, Punjab (Pakistan) | All women who had a pregnancy or child <2 years in intervention sites were eligible. | India – most caregivers (>95%) received at least one home visit throughout intervention but only 30% [range: 20-45%]) received visit in the last month. At 18 months, 19% of mothers received 60% or more of expected visits.  Pakistan – most caregivers received at least one home visit in past month (92% [range: 78-98%]) | In both sites, caregivers (including fathers and grandmothers) reported that visits had increased the amount of attention and time they gave their child, but families rarely reported that they had done the play activities routinely and frequently. | Fathers and grandmothers |
| †Thorne 2014, Kenya, Tanzania*, Uganda (*Aga Khan Foundation*) – ***training report*** | Urban and rural, Mombasa, Zanzibar, Kampala | Mothers of children < 3 years old. At-risk (e.g., malnourished, HIV exposed) and non-at-risk children |  |  | Fathers were included in group sessions and home visits in some settings |
| Yousafzai et al., 2014, 2015, 2018 Pakistan* | Rural, Sindh province | Mothers of infants aged 0-24 months old | N=3550 caregiver-child dyads were exposed to intervention/service. N=1489 were evaluated. 75% of households received home visits; 31% of all female caregivers participated in group meetings | Families reported many perceived benefits for their young children and their own emotional wellbeing | Grandmothers and fathers were welcome to group sessions (38% attended one or more sessions in combined arm, 28% in CCD arm) |
| Holding, 2015, Mali* (*UNICEF, Handicap International, BØRNEfonden*) | Urban and rural, Bamako and Sikasso | Mothers of young children |  |  | Fathers |
| PATH, 2015, Mozambique* (*PATH*) | Urban, Maputo province | Mothers of children at the health facility for child health visits |  | Playboxes led to a perception of reduced waiting time and increased client satisfaction in the health facilities |  |
| Rockers et al., 2016, Zambia | Rural, Chomba and Pemba districts in Southern province | All female caregivers with children < 5 years old were invited to participate. Evaluation was only conducted on female caregivers ≥15 years old with a child aged 6-12 months at enrollment | N=526 were evaluated. 68% of households were visited at least 20 times. On average, caregivers received 19 home visits and attended 14 group sessions. Most caregivers (89%) attended ≥10 sessions and only four caregivers (<2%) attended <5 sessions. Average attendance at group sessions was ~10 |  |  |
| Bayitondere et al., 2018, Rwanda* | Rural, Southern Kayonza district | Caregivers (70.6% mothers) of children presenting for care at the pediatric development clinic |  |  | Fathers and grandmothers |
| Fisher et al., 2018, Vietnam* – ***study protocol*** | Rural, Ha Nam province | All women aged ≥18 years, pregnant, and <20 weeks’ gestation living in selected communities. Exclusion criteria: women who have a cognitive disability (as determined by local commune health station staff) or other serious physical disabilities which prohibit attendance |  |  | Family members will be involved in the post-birth home visit |
| †Gladstone et al., 2018, Malawi* | Urban and rural, southern Malawi | Parents of children aged 0-2 years | N=60 caregiver-child dyads were exposed to intervention/service. Average group session had 6.3 women (range = 3-10). Mothers attended, on average, six groups out of ten | Barriers to attendance included funerals, need to work and farm, and need to get governmentally provided funds |  |
| †Lynch et al., 2018 and Gladstone et al., 2017, Malawi* | Urban and rural, southern Malawi | Caregivers of children aged 0-7/8 years who are registered as blind or with a severe visual impairment and not attending primary school. Also included children with other disabilities (e.g., cerebral palsy, paraplegia) | N=30 caregiver-child dyads were exposed to intervention/service. | In-depth interviews (n=20) and focus groups (n=6): caregivers reported benefits from programme and indicated that they felt supported with the use of the counselling cards and involvement of community workers. | Other adults who cared for the child were invited to participate |
| †Rosales et al., 2019 and World Vision International 2017, Armenia (*World Vision*) | Rural, Gegharkunik province | Caregivers of children <23 months old (including children with disabilities) | N=1300 caregiver-child dyads were exposed to intervention/service. 44% retention |  | Fathers and grandmothers |
| UNICEF 2019, Paraguay* (*UNICEF*) | Urban, Asunción, Atyra, and Coronel Oviedo | Services targeted different beneficiaries:  -Vulnerable families (based on low-income, place of residence, and lack of access to education and other services) with children aged 0-3 years  -Children aged 6 months-4 years  -Incarcerated mothers and children aged 0-4 years  -Families living in extreme poverty | More than n=3000 caregiver-child dyads were exposed to these interventions/services. |  | Fathers were included in some programmes |
| Zhou et al., 2019, China* | Rural, Guizhou and Shanxi provinces | Mothers of children aged 0-35 months | Intervention/service was available to all children aged below 3 years in intervention villages. N=2953 caregiver-child dyads were evaluated. 66% of families attended ECD center activities of play and reading at least once in month prior to endline; 88% attended clinic. |  |  |
| Barnhart et al., 2020, Rwanda | Rural, Nyanza, Ngoma, and Rubavu districts | Parents of children 6-36 months from lowest locally-defined socioeconomic stratum | N=38 caregiver-child dyads were evaluated. | 79-100% satisfaction on different aspects of programme | Fathers may have been present during home visit |
| Betancourt et al., 2020, Rwanda | Rural, Nyanza, Ngoma, and Rubavu districts | Parents of children 7-36 months from lowest two locally-defined socioeconomic strata | N=1049 caregiver-child dyads were evaluated. | 97% of caregivers were satisfied with the programme | Fathers |
| Rao et al., 2020 and Chan et al., 2021, Bhutan* (*UNICEF*) | Three districts each from the Western, Central, and Eastern regions | Caregivers (mostly mothers) of children aged <2 years | N=425 caregiver-child dyads were evaluated. |  |  |
| Shah et al., 2020, India | Rural, Karnataka state | Primary caregivers of infants aged 6 weeks to 6 months presenting for well-child visit | N=47 caregivers were exposed to intervention/service. | High levels of caregiver acceptance and enactment of play messages. Caregivers perceived a need for the intervention/service and found the content and structure useful. Suggestions for intervention/service improvement included adding more visual content and disseminating information to non-parental caregivers and fathers. |  |
| Shi et al., 2020, China | Urban, Zhanlanlu street, Xicheng district, Beijing | Caregivers of infantes aged ~2-months meeting following criteria: term infants aged 1-2 months w/ birth weight >2500g and caregiver agreement to participate. Exclusion criteria: preterm infants, multiple births (e.g., twins), and infants w/ birth asphyxia, deformity, or mental retardation | N=140 caregiver-child dyads were evaluated. All intervention caregivers (n=82) attended the first parenting training session and 73 (89%) attended the second session. 47 telephone parenting guidance sessions were conducted. |  |  |
| Xu et al., 2020, China – ***study protocol*** | Rural, Shanxi province | Caregivers of children aged 2-30 months |  |  |  |
| †African Population Health Research Centre, 2021, Kenya* (*PATH*) | Siaya county, western Kenya | Caregivers of children 0 to ~30 months, including children with disabilities and children exposed to HIV | N=616 caregiver-child dyads were evaluated. | Caregivers were satisfied with the quality of service delivery and indicated that the intervention improved their childcare skills by providing them with opportunities to learn new information regarding children’s care and development. Also indicated that playbox sessions had made long waiting times more bearable. | Male caregivers |
| †Aga Khan University and Aga Khan Foundation, 2021, Kenya* (*Madrasa Early Childhood Programme*) | Rural, Vipingo and Gongoni in Kilifi South sub-county | Underprivileged communities |  | Delivery agents indicated that mothers reported positive feelings and responses from their unborn children and that fathers were happy to better connect with their children. | Male caregivers |
| Aga Khan University and Aga Khan Foundation, 2021, Syria (*AKDN*) | Urban, Damascus, Hama, Tartous | Caregivers of children <5 years |  | Delivery agents reported that parents had greater confidence in the services and the staff because parents specifically ask about their child’s development and no longer skip follow-up visits as they used to do. |  |
| †Aga Khan University and Aga Khan Foundation, 2021, Tanzania* (*Madrasa Early Childhood Programme and Elizabeth Glaser Pediatric AIDS Foundation*) | Zanzibar and Southern Tanzania | Caregivers of children 0-3 years, including children affected by or infected with HIV/AIDS and children with disabilities |  |  | Fathers |
| †Aga Khan University and Aga Khan Foundation, 2021, Uganda* (*Madrasa Early Childhood Programme and Save the Children*) | West Nile and Central regions | Caregivers of children 0-3 years, including teenage parents, HIV+ parents, parents struggling with alcoholism |  |  | Fathers |
| Akhmadi et al., 2021, Indonesia* – ***training evaluation*** | Rural, Kulon Progo district, Yogyakarta province | *Training of healthcare volunteers* |  |  |  |
| Dovel et al., 2021, Malawi* (*Partners in Hope*) | Central Malawi | HIV-positive mothers of infants <24 months receiving prevention of mother to child transmission care | In-depth interviews were conducted with n=29 mothers. | Women enjoyed being in the programme. Desired sessions to help them cope as mothers living w/ HIV. Participants believed that the programme should have explicit, repetitive lessons on why certain activities (e.g., playing with and talking to infants) are needed and how to play and talk with infants | None |
| Jensen et al., 2021, Rwanda | Rural, Nyanza, Ngoma, and Rubavu districts | Primary caregivers who (1) were eligible for either the classic public works, which provided cash for intensive manual labour, or the newer expanded public works, which provided cash for more flexible, less strenuous work and thus all belonged to the most extreme level of poverty in the government’s household-ranking system, Ubudehe 1; (2) had one or more child(ren) aged 6–36 months and (3) were willing to engage in a home-visiting intervention | N=1084 caregiver-child dyads were evaluated. |  | Home visits involved participation of female and male caregivers (as present). Other caregivers and children in household were welcome to participate |
| Jeong et al., 2021, and Bliznashka et al., 2022 Mozambique* (*PATH*) – ***qualitative evaluation*** | Rural, Monapo district, Nampula province | Eligibility criteria for caregivers  included: primary caregiver of a child <2.5 years of age who resided in the same household as the child, caregiver’s household was located within the  catchment area of the health facility, and caregiver visited the health facility for child health service in the past month (or that day for exit interviews). | In-depth interviews were conducted with n=36 caregivers, n=15 facility-based health providers, n=12 community-based health providers, and n=10 district stakeholders. | Caregivers were satisfied with messages and services received. They valued the nutrition and parenting messages and the positive interpersonal counseling skills of facility providers. Among those who engaged with media, most were satisfied and found media-based information easy to understand and informative. Among those who engaged with both facility and community-based services, caregivers thought messages were coordinated and consistent between the two services. When asked to compare facility and community services, caregivers preferred facility counseling because facility providers had more patience, better interpersonal skills, and explained content more clearly. | Fathers were encouraged to participate in some services |
| UNICEF 2021, Belize and Anguilla* (*UNICEF and PAHO/WHO*) | Rural and urban, Toledo district and Belize city  Anguilla | Different services  -National MCH and CHW programmes – mothers of young children  -Roving caregivers programme – caregivers of children <3 years who lack access to formal early childhood education services  -Response to Hurricane Irma – caregivers of children 0-3 years |  |  | Fathers and other family members were involved in some services |
| UNICEF 2021, the Dominican Republic* (*UNICEF and PAHO/WHO*) | Urban, Santo Domingo | Different services:  -Kangaroo Mother Care – caregivers (mostly mothers) of premature or low birthweight newborns  -Mother and Child Pastoral (PMI) – caregivers of children <5 years from vulnerable populations (e.g., children w/ Zika syndrome-related microcephaly and/or congenital anomalies, children w/ disabilities) |  |  | Fathers and other family members |
| UNICEF 2021, El Salvador* (*UNICEF and PAHO/WHO*) | Rural and urban, Ahuachapán, Cabañas, Chalatenango, Cuscatlán, La Libertad, La Paz, San Salvador, and San Vicente and Sonsonate departments | Different services:  -At-risk populations – pregnant and postpartum women, newborns, children <5 years, people with disabilities  -caregivers of children with inherited or acquired physical, cognitive, or mixed disabilities  -Family Circles – pregnant women, families, caregivers, and children <5 years  - Comprehensive early childhood care programme – caregivers of children aged 3 months to 7 years |  |  | Fathers and other family members were involved in some services (e.g., Family Circles) |
| UNICEF 2021, Peru* (*UNICEF and PAHO/WHO*) | Rural and urban, Loreto, Ucayali, and Huancavelica regions and Carabayllo district in Lima | Different services:  -Growth and Development Monitoring (CRED) – caregivers of children <5 years  -Cuna Más – caregivers of children <3 years living in areas with widespread or extreme poverty  -Early intervention programme – caregivers of children <3 years with developmental delays and/or disabilities |  |  |  |
| †UNICEF 2021, Tunisia, Iran, Egypt* (*UNICEF*) | Alexandria and Qalubia (Egypt), Mednine, Haffouz, Melassine, and Balta (Tunisia) | Egypt – caregivers of 0-5-year-old children  Iran – all primary caregivers of children <3 years (including pregnant women), including children with disabilities  Tunisia – pregnant women and caregivers of 0-5-year-old children |  |  | Fathers (Tunisia) |
| †Antelman et al., 2022, Tanzania (*Elizabeth Glaser Pediatric AIDS Foundation*) | Rural, Tabora, central-western Tanzania | Adult primary caregivers (97.9% mothers) of children 0-24 months | N=1248 caregiver-child dyads were exposed to intervention. N=1004 were evaluated. Fifty-five to seventy-five percent of caregivers reported hearing ECD radio message. Caregivers received a median of 8 visits from delivery agents during intervention period, 84% received 6 or more visits. |  | None; only one adult caregiver per household was recruited to study |
| Bahari Gharehgoz et al., 2022, Iran* | Tabriz | Primary caregivers (mothers and fathers) of children 4-36 months old with a developmental delay (-1 or -2 SD score on ASQ or diagnosis as high-risk child by health professional) | N=50 caregiver-child dyads were exposed to the intervention and evaluated. |  |  |
| Bemanalizadeh et al., 2022, Iran* | Isfahan | Pregnant women (18-45) years with a healthy fetus | N=210 caregiver-child dyads were exposed to the intervention. N=181 were evaluated. |  |  |
| †Ummeed 2022, India (*Ummeed*) | Rural and urban, Assam, Gujarat, Himachal, Maharashtra, Meghalaya, and Uttarakhand states | Different services:  -Early Childhood Champions – primary caregivers of children 0-3 years in low-resource communities.  -Child Development Aide – primary caregivers of 0–6-year-old children with disabilities | N=6504 caregiver-child dyads exposed to Early Childhood Champions. N=3588 caregiver-child dyads exposed to Child Development Aide. | Caregivers reported increased awareness of inclusive ECD. Some caregivers became inclusive ECD champions in their communities, advocating for early identification and intervention of developmental delay and disability. |  |
| Jeong et al., unpublished manuscript, Tanzania* (*Global Communities*) | Rural, Musoma and Butiama districts, Mara region | Eligible families met the following criteria: 1) household has a child under 18 months of age at the time of enrollment; 2) fathers are present in households with mother and index child for at least ten months out of the year; 3) mother, father, and index child plan to reside in study area over the course of study period; and 4) both parents provide informed consent to participate in the study | N=960 families exposed to intervention, N=815 evaluated. Average attendance at group sessions was 71-75% across study arms, with lower rates among fathers (70%) than mothers (74%). Average attendance at home visits was 87-91% (87% among fathers, 90% among mothers) across study arms. | Qualitative data were collected but have not yet been published | Fathers |
| †McHenry et al., unpublished manuscript, Kenya* | Rural, Uasin Gishu County | Eligible children met the following inclusion criteria: (1) participation in the NeuroDEV study (of HIV-unexposed, HIV-exposed, and HIV-infected children; (2) a composite score ≤ 85 on the Bayley-3; (3) ages between 18-24 months; (4) lives within a household that speaks Kiswahili or English. Each eligible child’s caregiver also had to meet the following inclusion criteria: (1) primary caregiver a child who participated in the NeuroDEV study; (2) age 18 years or older; and (3) fluent in Kiswahili or English. | N=31 caregiver-child dyads were evaluated. | Caregivers enjoyed receiving sessions in a group setting and reported benefitting from the support they received from others within the group. They also identified sessions on managing stress, disciplining techniques, and how to invest and plan for the future as the most useful. Overall, caregivers expressed enjoyment in reading and playing with their children and reported progress in their child’s development over time. | None |

Note: Missing information (e.g., empty cells) indicates that data were not clearly provided

†Extracted data were supplemented by information provided in key informant interview(s)

*CCD-based service

ASQ = Ages and Stages Questionnaire. ECD = Early childhood development

^1^For studies which report the reach (i.e., number of caregiver-child dyads who received the intervention or service) of a given intervention/service, these data are included in the description of beneficiaries.

**Table S3.** Characteristics and training of delivery agents in interventions and services using the Care for Child Development Package

| **Author, year, country (*implementing partner*)** | **CCD delivery agents: sex, background, compensation** | **Training: duration, trainers, learning methods, refreshers, process questions, evaluation** | **Supervision and monitoring** | **Integration into existing government service** |
| --- | --- | --- | --- | --- |
| Ertem et al., 2006, Turkey* | Pediatricians |  | Researcher monitored clinic visit using Physician Counselling Skills Scale | Integrated into health sector: pediatrician’s responsibilities |
| Jin et al., 2007, China* | Health professional | Trained in four-step communication |  |  |
| Ertem et al., 2009, Turkey – ***training evaluation*** | Male and female (89%) general practitioners and nurse-midwives w/ >5 years’ experience. Only 5% had received any formal training in ECD | Five-day training by academics. Educational material included textbook, trainer’s guide, CD w/ presentations and video clips, and case scenarios. Delivery agents practiced skills at healthcare centres (active learning). Assessed knowledge on child development and perceived competence pre- and post-training. |  | Integrated into health sector: GPs worked in clinics and nurse-midwives conducted home visits |
| Engle et al., 2011, Kazakhstan, Kyrgyzstan, and Tajikistan* | Male and female clinic health workers (nurses, pediatricians), community health workers, and volunteers (only in Tajikistan) | Kazakhstan: Five-day training, included guided practice. Kyrgyzstan: 3-day + 2-hour training. Tajikistan: 8-hour training + 4-hour practical session. |  | Integrated into health sector |
| Jones, 2012, Mozambique* (*Aga Khan Foundation*) | Delivery agents with agriculture, health, and education backgrounds | 2-day training | Delivery agents were observed during some home visits |  |
| †Lingam et al., 2014 and SPRING Trial Team personal communication, India and Pakistan* | India – Kilkaari workers (community-based agents recruited by an NGO)  Pakistan – Lady Health Workers (LHWs) | Train-the-trainer model: ECD expert trained SPRING Trial supervisors (female social science graduates), who then went on to train delivery agents. Each training lasted 8 days. | Delivery agents received monthly group supervision from Lady Health Supervisors (Pakistan) and SPRING Trial supervisors (India and Pakistan), as well as one-on-one field supervision from SPRING Trial supervisors using an observation checklist (India and Pakistan). Supervision consisted of peer-learning, problem-solving, skill development, peer support, and supportive feedback. Delivery agents’ knowledge gain was assessed pre- and post-training (increased from 70 to 85% in India and 50 to 91% in Pakistan). Quality of training was also assessed through training attendance logs and satisfaction survey. | Pakistan – integrated into health sector: Lady Health Worker programme |
| †Thorne 2014, Kenya, Tanzania*, Uganda (*Aga Khan Foundation*) – ***training report*** | Different services: volunteer community health workers w/ at most high-school diploma (Kenya), preschool teachers and healthcare workers (Tanzania, Uganda) | 4-5-day training by UNICEF technical support (train-the-trainer model). Those trained were community health extension workers (Kenya) and other supervisors/mentors (Tanzania, Uganda) who got two week’s training (1 week on CCD, 1 week practicing training others) Refresher courses were held four times a year. | Delivery agents had ~monthly meetings with master trainers and one-time on the job-supervision using observation checklists by them. There was some peer support by working in pairs. | Integrated into health and education sectors |
| Yousafzai et al., 2014, 2015, 2018 Pakistan* | Lady Health Workers (LHWs). Paid US$ 85/month by government | Three days for CCD package, two days for nutrition package, and five days for combined package (included active learning). Trainers were social workers/teachers with Bachelors (train-the-trainer model). Trainers were trained by researchers. Refreshers were held every 6 months. | Trainers also supervised, monitored (using supervisory checklists), and mentored delivery agents: on-the-job supervision and coaching twice a month. Evaluation of delivery agents’ program acceptance. | Integrated into health sector: Lady Health Worker programme |
| Holding, 2015, Mali* (*UNICEF, Handicap International, BØRNEfonden*) | Social workers, community workers, and animators | Multi-country train-the-trainer session referenced |  |  |
| PATH, 2015, Mozambique* (*PATH*) | Community activists |  |  | Integrated into health sector: health facilities |
| Rockers et al., 2016, Zambia | Mothers selected by community members delivered group sessions. Child development agents (paraprofessionals) delivered home visits | Five-day training for child development agents (train-the-trainer model). They trained ‘head mothers’ ahead of each session. | Child development agents provided resources for ‘head mothers’ before each session, monitored attendance at group sessions, and completed checklists on home visits. | Intervention was not integrated into any existing government services |
| Bayitondere et al., 2018, Rwanda* | Community health workers |  |  | Integrated into health sector: community health workers’ services |
| Fisher et al., 2018, Vietnam* – ***study protocol*** | Female members of local women’s union with experience conducting community programmes. Community health workers and kindergarten teachers co-facilitated some sessions | Trained at the start of each module |  | Integrated into social sector: Women’s Union |
| †Gladstone et al., 2018, Malawi* | Male and female health surveillance assistants w/ 12-week training in role | Trained by staff from Ministry of Health and Nutrition. Sessions were audio-recorded to gain feedback on materials and training. Training included 2-3 days of practicing with caregivers and children in healthcare settings. | Research assistants from study team conducted supervisory visits using observation checklists to monitor fidelity and deliver. | Integrated into health sector: health surveillance assistants |
| †Lynch et al., 2018 and Gladstone et al., 2017, Malawi* | Male and female community workers (professionals including child protection officers, specialist teachers, and community-based rehabilitation workers). Paid US$4.20/visit | Train-the-trainer model: Principal investigators (researchers with ECD background and CCD training) trained delivery agents over 3 days, including a 1-day feasibility study. | Assessed delivery agents’ competency post-training. Delivery agents gave anonymized feedback on training. Research assistants supervised and monitored delivery agents | Intervention was not integrated into any existing government services |
| †Rosales et al., 2019 and World Vision International 2017, Armenia (*World Vision*) | School and kindergarten teachers, nurses, and social workers w/ university degrees | 3-day training of stakeholders, ministry of health staff, and technical experts by World Vision (ECD experts) and Global Centre (technical lead for maternal, newborn, and child health) staff (train-the-trainer). Delivery agents were trained for 3-4 days. Refresher sessions were held. | Checklists were used by supervisors to assess delivery. | Integrated into health and social sectors |
| UNICEF 2019, Paraguay* (*UNICEF*) | Different services:  -Male and female local teachers (“backpack teachers”) w/ >3 years of teacher training  -Former preschool or grade school teachers w/ >3 years of teacher training  -Former early childhood and grade school teachers  -Community volunteers: local mothers who are functionally literate | Workforce and officials received CCD master training (train-the-trainer model). | -“Backpack teachers” were supervised monthly by department-level Ministry of Education and Culture staff (qualified teachers w/ university degrees in psychology, pedagogy, educational sciences, etc.) w/ ~3-4 years’ experience in supervisory roles  -Former teachers were supervised 3x annually by Ministry of Education and Culture staff person with university degree in psychology  -Former teachers were supervised by prison staff with education background  -Community volunteers were supervised weekly by Ministry of Social Development community guides (high school educated and some community work experience) | Integrated into various sectors: Ministry of Education and Culture, Ministry of Social Development, Ministry of Justice |
| Zhou et al., 2019, China* | Volunteers w/ minimum 8 years of education, facility-based health providers, and professionals with expertise in maternal and child health |  | Bimonthly supervision | Integrated into health sector |
| Barnhart et al., 2020, Rwanda | Male and female community volunteers who were literate in Kinyarwanda. Paid 26 800 RWF/month | 2-week training | Supervisors were bachelor-level staff members w/ training in clinical psychology or social work. Supervised delivery agents every other week, including shadowing, and provided feedback. Also held monthly group sessions w/ delivery agents | Intervention was not integrated into any existing government services |
| Betancourt et al., 2020, Rwanda | Male and female community-based volunteers | 3-week (120 hours) training by researchers and graduate-level experts. Training included role-play-based learning and active coaching practice. Received monthly in-person group refreshers by supervisors. | Supervisors were graduate-level experts who provided 3 weeks of in-person supervision, weekly phone supervision (12 hours), and weekly in-person peer support groups. | Intervention was not integrated into any existing government services |
| Rao et al., 2020 and Chan et al., 2021, Bhutan* (*UNICEF*) | Health assistants with minimum 12 years of schooling, on average. |  | Supervisors were health officers with university degree | Integrated into health sector: clinics and other health-related work |
| Shah et al., 2020, India | Pediatrician, physical therapist, and research assistant | 2-day training (8 hours) by professionals. Included active learning methods (demonstration, practice, role plays, discussion). Assessed delivery agent fidelity to intervention during training. |  | Integrated into health sector: immunization clinics |
| Shi et al., 2020, China | Child development experts. Received US$ 130.23 per parenting training session. |  | Staff in Department of Child Healthcare in community health centre at Zhanlanlu Hospital were responsible for supervising and managing intervention | Integrated into health sector |
| Xu et al., 2020, China – ***study protocol*** | Health and family planning personnel, experienced volunteers, female caregivers, kindergarten teachers. Paid ~450 Chinese Yuan/month. | 2-week training. Delivery agents received refresher training sessions to answer frequently asked questions. |  | Intervention was not integrated into any existing government services |
| †African Population Health Research Centre, 2021, Kenya* (*PATH*) | Clinical service providers (nurses, clinical officers) and community health volunteers | Train-the-trainer model: PATH ECD staff trained health management team who then trained the delivery agents. Trainings lasted 3-5 days and included active learning sessions of practicing with caregivers and children. | Delivery agent’s competencies and knowledge were assessed pre- and post-training. Health management team provided support supervision (including CCD observation checklist), mentorship, and on-the-job training for delivery agents. Refresher trainings were held. | Integrated into health sector: Ministry of Health |
| †Aga Khan University and Aga Khan Foundation, 2021, Kenya* (*Madrasa Early Childhood Programme*) | Community health volunteers and community health assistants | Train-the-trainer model: CCD consultants trained CHVs w/ basic educational level (5 days). These master trainers then went on to train delivery agents (5 days). | Supervisors/mentors were Madrassa Early Childhood Programme staff. They observed delivery agents during implementation, held one-on-one and group mentor sessions, and ran a 2-day refresher training. CHVs reported weekly progress to health facilities. | Integrated into health sector: community health strategy (e.g., MCH, maternity, and nutrition departments) |
| Aga Khan University and Aga Khan Foundation, 2021, Syria (*AKDN*) | Health workers (nurses, pediatricians, etc.) and health educators (community-level health workers) | Train-the-trainer model: health worker supervisors/managers and qualified health workers received 5-day training and then went on to train other delivery agents over 5 days. | Trainees were tested before and after training on knowledge, attitudes, and practices regarding child health and development. Average score increased from 45% pre-training to 79%. Health worker supervisors/managers observed delivery agents counselling families and provided feedback weekly. They completed weekly reports and submitted them to national authorities. | Integrated into health sector: Child wellbeing programme |
| †Aga Khan University and Aga Khan Foundation, 2021, Tanzania* (*Madrasa Early Childhood Programme and Elizabeth Glaser Pediatric AIDS Foundation*) | Community health workers and healthcare workers (e.g., doctors, nurses) | Train-the-trainer model: Initial 3-day training of regional and district-level government officials was conducted by master trainers from UNICEF and the Aga Khan Foundation. These master trainers then went on to train delivery agents. Master trainers and delivery agents did more practice sessions to enhance skills before programme roll-out. | Master trainers had follow-up session with trainers from Aga Khan Foundation and peer mentorship. Quantitative and qualitative data on implementation processes (i.e., training, delivery of home visits, clinic visits, and group sessions) were collected using Activity Report form. Delivery agents attended reflection meetings. CCD observation checklist was used to assess delivery agents’ counselling skills. Supervisors were sub-county community health officers. Delivery agents prepared reports on implementation (# of households visited, issues households are facing, advice given to caregivers) and shared them with their supervisors. | Integrated into health sector: Ministry of Health |
| †Aga Khan University and Aga Khan Foundation, 2021, Uganda* (*Madrasa Early Childhood Programme and Save the Children*) | Village health teams, counsellors, health workers, preschool teachers | Train-the-trainer model: Madrasa Early Childhood Programme (MECP) staff and individuals from district head offices were trained to be master trainers by CCD master trainers (an independent consultant and staff member of Aga Khan Foundation, Geneva) for 4 days. These in-country master trainers practiced for a month while receiving feedback from master trainers. They then went on to train delivery agents for 4-5 days. Training includes active learning component with trainees practicing with caregivers and children, including children with special needs. | In-country master trainers received refresher training. Delivery agents have pre- and post-training evaluation of ECD knowledge. They also fill out evaluation form to give their feedback on the training. Trained officers in charge of MECP ECD centres and health center representatives provided monitoring and supervision of village health teams. Village health teams prepared reports on implementation (# of households visited, issues households are facing, advice given to caregivers) and shared them with their supervisors. | Integrated into health, education, and social sectors: Ministry of Health, Ministry of Education and Sports, and Ministry of Gender and Social Development |
| Akhmadi et al., 2021, Indonesia* – ***training evaluation*** | Female healthcare volunteers, 70% completed junior high (grade 9) and 30% completed senior high. Had ~10-year experience in role. Unpaid position. | 2-day (10 hours) training by nurses (Masters/PhDs) from Gadja Mada University included watching videos, Q&A, role play, demonstrations with local toys, and active learning components. Assessed knowledge and attitudes about child development and self-efficacy to deliver messages to caregivers. | Supervisors were nurses at public health centres | Integrated into health sector: public health centres |
| Dovel et al., 2021, Malawi* (*Partners in Hope*) | Expert Clients: HIV-positive volunteer community members who provide counselling and support to HIV-positive individuals | 2-week training | Trained expert clients were responsible for ECD implementation at their facilities | Integrated into health sector |
| Jensen et al., 2021, Rwanda | Male and female community-based volunteers | 3-week (120 hours) training by researchers and graduate-level experts. Training included role-play-based and active learning. Received monthly in-person group refreshers by supervisors. | Supervisors were graduate-level experts who provided 3 weeks of in-person supervision, weekly phone supervision (12 hours), and weekly in-person peer support groups. | Intervention was not integrated into any existing government services |
| Jeong et al., 2021, and Bliznashka et al., 2022 Mozambique* (*PATH*) – ***qualitative evaluation*** | Different services:  - male and female health facility providers (maternal and child health nurses, preventive medicine technician, general medicine technician)  -male and female community health workers  Most delivery agents had completed secondary school. | Train-the-trainer model: PATH staff trained district Ministry of Health staff and partners who then trained health facility providers and community health workers. Health facility providers received ongoing training through on-the-job supervision and mentoring. Community health workers received refresher trainings. | PATH staff and existing supervisors in health facilities and community health structures provided regular (~monthly) supervision. | Integrated into health sector: health facilities and community health worker system |
| UNICEF 2021, Belize and Anguilla* (*UNICEF and PAHO/WHO*) | Different services  -National MCH and CHW programmes – health providers  -Roving caregivers programme – community health workers (volunteer community members) | Train-the-trainer model: district- and national-level officers and technical personnel were trained to become master trainers by one of CCD’s principal creators and ECD experts. Master trainers then trained service providers in health, human development, and education sectors for 2 days. | Master trainers reported that training of trainer approach enabled progress in ECD policy developments. CHWs are supervised by Health Education and Community Participation Bureau (and health nurse or public health nurse in rural communities). Care for Child Development monitoring and evaluation surveillance system was developed to outline and monitor CCD implementation. | Integrated into health and human development sectors in Belize: Ministry of Health and Wellness; Ministry of Human Development, Families and Indigenous Peoples’ Affairs |
| UNICEF 2021, the Dominican Republic* (*UNICEF and PAHO/WHO*) | Different services:  -Kangaroo Mother Care – nursing, neonatology, and psychology staff and resident doctors  -PMI – volunteer community agents | CCD training by UNICEF and PAHO/WHO in Honduras and Paraguay for high-level government officials. Followed by in-country CCD intersectoral training the trainer workshop for national trainers and technicians from government agencies and NGOs. These trainers went on to train their colleagues in their respective entities. | Delivery agents reported that training encouraged changes in team attitudes and practices in support of ECD. PMI developed tools to monitor implementation and activities carried out during home visits. | Integrated into health and education sectors: Ministry of Public Health and Ministry of Education (through work of PMI, a social action organization) |
| UNICEF 2021, El Salvador* (*UNICEF and PAHO/WHO*) | Different services:  -health promoters  -maternity waiting home technical officials and front-line personnel  -rehabilitation center staff – educators, physical, speech, and music therapists, psychiatrists, pediatricians, other doctors  -Family Circles – staff members of Technical Assistance for Early Childhood  -Parenting workshops – teachers  -Comprehensive early childhood care programme – social workers, psychologists, child educators | Week-long CCD training by UNICEF and PAHO/WHO in Honduras for high-level government officials. Followed by 5-day in-country train-the-trainer session (representatives of education, health, and rehabilitation services). Delivery agents were trained for 3 days or less. Post-training interviews show that trainees appreciated content, innovative approach, and multidisciplinary and multisectoral nature of training. |  | Integrated into health and education sectors: Ministry of Health; Ministry of Education, Science and Technology |
| UNICEF 2021, Peru* (*UNICEF and PAHO/WHO*) | Different services:  -Growth and Development Monitoring (CRED) – trained nurse or medical staff  -Cuna Más – community agents with support from nutritionists, doctors, education technicians, as necessary and family guides (local women)  -Early intervention programme – professional tutors (teachers, doctors, and psychologists specialized in early care of children with disabilities or at risk of developing disabilities) | UNICEF-led 4-day Basic CCD course for national trainers (government officials and NGO personnel) who became Core Group of National Trainers. This training included field trips to a hospital, health centre, and a daycare centre. UNICEF Peru and a local university (UPCH) designed a four-module hybrid CCD diploma course for public officials and delivery agents: each module included 1 week of in-person classes followed by remote or virtual support through university’s distance learning platform. Each participant completed 120 hours of theory and 224 hours of practice. Those who completed course then went back to their service and trained their fellow delivery agents. | UNICEF Peru team monitored the training the diploma course participants administered when they returned to their respective services. The diploma course helped change logic of work and training in various gov’t sectors. National trainers indicated that Basic CCD course enabled them to learn about benefits of CCD and its relevance to ECD initiatives. Observation guide was developed to monitor performance of CRED nurses. | Integrated into health, education, and social sectors: Ministry of Health; Ministry of Development and Social Inclusion; Ministry of Education |
| †UNICEF 2021, Tunisia, Iran, Egypt* (*UNICEF*) | Egypt – primary health care delivery agents  Iran – doctors (pediatrician, child psychiatrist), nurses, midwives, and orphanage personnel who work and provide care to children  Tunisia – doctors (pediatrician, child psychiatrist), nurses, midwives, social workers, and nursery teachers | Train-the-trainer model:  Egypt – IMCI facilitators (doctors, nurses) were trained by UNICEF consultants for 4 days, which included an active learning component. These master trainers then went on to train delivery agents.  Iran – experts were trained by UNICEF consultants for 5 days. These master trainers will go on to train delivery agents.  Tunisia – health professionals and staff of Positive Parenting Programme were trained by UNICEF consultants. These master trainers then went on to train delivery agents. | Assess delivery agent knowledge and skills pre- and post-training. District health officers supervise delivery agents (~quarterly) and use checklists to monitor delivery. | Egypt – integrated into health sector: Ministry of Health and Population  Iran – integrated into health, child protection/social, and education sectors: Ministry of Health and Medical Education  Tunisia – integrated into health child protection/social, and education sectors: Positive Parenting Programme |
| †Antelman et al., 2022, Tanzania (*Elizabeth Glaser Pediatric AIDS Foundation*) | Community health workers | Train-the-trainer model: individuals at Elizabeth Glaser Pediatric AIDS Foundation were trained for 6 days by Aga Khan. They then trained delivery agents on CCD and how to use video job aids for 2 days | Fidelity monitoring data were collected monthly to document completion of home visits. | Integrated into health sector |
| Bahari Gharehgoz et al., 2022, Iran* | Intervention was provided by participants’ health and medical services |  |  |  |
| Bemanalizadeh et al., 2022, Iran* | Pediatric neurologist |  |  |  |
| †Ummeed 2022, India (*Ummeed*) | Mostly female community health workers from community-based organizations with education levels up to 10^th^ grade | Train-the-trainer model: Ummeed staff were trained in CCD by one of CCD’s principal creators and an academic ECD expert. They then went on to train delivery agents. Training included active learning components.  -Early Childhood Champions – community health workers receive training for 4 modules (4 days for each module) over a year  -Child Development Aide – community health workers receive 6-month training. | Assess delivery agent knowledge and skills pre- and post-training. Delivery agents receive mentorship from Ummeed staff and are supervised by project supervisors from their parent organization. Training also consists of module specifically for these project supervisors. CCD’s observation checklist is used to monitor delivery agents. |  |
| Jeong et al., unpublished manuscript, Tanzania* (*Global Communities*) | Male and female community health workers | Train-the-trainer model: Study investigators trained supervisors (Global Communities staff hired for intervention) for 3 weeks on all intervention packages. Supervisors then trained delivery agents for 13 days, including 3 days dedicated to CCD training. Training included active learning components. | Supervisors led quarterly 3-day refresher trainings. Supervisors routinely observed delivery agents (10-15% agents observed monthly) using a supportive supervision checklist to document fidelity and provide coaching. Delivery agents were mentored in small groups on a quarterly basis. | Integrated into health sector |
| †McHenry et al., unpublished manuscript, Kenya* | Female research assistant with background as clinical officer | Train-the-trainer model: Principal investigator of study attended UNICEF training workshop then went on to train delivery agent for 1 week. | Principal investigator supervised delivery agent by revieing and giving feedback on each session | Intervention was not integrated into any existing government services |

Note: Missing information (i.e., empty cells) indicates that data were not clearly provided

†Extracted data were supplemented by information provided in key informant interview(s)

*CCD-based service

CHVs = community health volunteers. ECD = early childhood development. IMCI = integrated management of childhood illnesses. MCH = maternal and child health. NGO = non-governmental organization.

**Table S4.** Evaluation of the impact of interventions and services using the Care for Child Development Package on child and caregiver outcomes

| **Author, year, country (*implementing partner*)** | **Study design** | **Sample size** | **Caregiving outcomes** | **ECD outcomes** | **Other child/caregiver outcomes** |
| --- | --- | --- | --- | --- | --- |
| Ertem et al., 2006, Turkey* | Non-randomized | I: 130  No I: 129 | HOME: ns |  | Acute illness: ns |
| Jin et al., 2007, China* | Cluster-RCT | I: 50  C: 50 | KAP: not reported | Gesell Development Schedules: I>C |  |
| Ertem et al., 2009, Turkey – ***training evaluation*** |  |  |  |  |  |
| Engle et al., 2011, Kazakhstan, Kyrgyzstan, and Tajikistan* | Non-randomized | Tajikistan – n=118  Kyrgyzstan – n=112 | FCI: ns | Tajikistan – ASQ: I>C (gross motor, communication, personal social), ns (problem-solving, fine motor)  Kyrgyzstan – ASQ: I>C (gross motor, communication), ns (problem-solving, fine motor, personal social) |  |
| Jones, 2012, Mozambique* (*Aga Khan Foundation*) |  |  |  |  |  |
| †Lingam et al., 2014 and SPRING Trial Team personal communication, India and Pakistan* | Cluster-RCT (results not yet published) |  |  |  |  |
| †Thorne 2014, Kenya, Tanzania*, Uganda (*Aga Khan Foundation*) – ***training report*** |  |  |  |  |  |
| Yousafzai et al., 2014, 2015, 2018 Pakistan* | Cluster-RCT | I (CCD): 383  I (nutrition): 364  I (combined): 374  C: 368 | HOME: I>C  Knowledge and practices about ECD: I>C  OMCI: I>C | Bayley: I>C (cognition, language, motor); ns (socio-emotional) | SRQ-20: I<C |
| Holding, 2015, Mali* (*UNICEF, Handicap International, BØRNEfonden*) |  |  |  |  |  |
| PATH, 2015, Mozambique* (*PATH*) |  |  |  |  |  |
| Rockers et al., 2016, Zambia | Cluster-RCT | I: 268  C: 258 | MICS: I>C | Intergrowth-Neurodevelopment Assessment tool: ns | Dietary diversity: I>C  SRQ-20: ns  Illness in past 2 weeks: ns  Weight-for-age: ns  Height-for-age: ns |
| Bayitondere et al., 2018, Rwanda* |  |  |  |  |  |
| Fisher et al., 2018, Vietnam* – ***study protocol*** |  |  |  |  |  |
| †Gladstone et al., 2018, Malawi* |  |  |  |  |  |
| †Lynch et al., 2018 and Gladstone et al., 2017, Malawi* | Feasibility pilot with no assessment | I: 30 |  |  |  |
| †Rosales et al., 2019 and World Vision International 2017, Armenia (*World Vision*) | Non-randomized | I: 140  No I: 130 | Brigance Parent-Child Interaction Scale: I>No I | Bayley composite: I>No I (comparing those with IQ>85)  Bayley cognitive, language, motor: ns | Dietary diversity: I> No I |
| UNICEF 2019, Paraguay* (*UNICEF*) |  |  |  |  |  |
| Zhou et al., 2019, China* | Non-randomized pre-/post-test | I: 1468  No I: 1485 |  | ASQ: I>No I |  |
| Barnhart et al., 2020, Rwanda | Cluster randomized pilot | I (ECD + public works programme): 19  I (public works only): 19 | HOME: I (ECD + public works programme) > I (public works only)  OMCI: I (ECD + public works programme) > I (public works only)  MICS: I (ECD + public works programme) > I (public works only) | MDAT: ns  ASQ: ns | Dietary diversity: I (ECD + public works programme) > I (public works only)  Shared decision-making: I (ECD + public works programme) > I (public works only)  Family conflict: I (ECD + public works programme) < I (public works only)  Illness past week: ns |
| Betancourt et al., 2020, Rwanda | Stratified cluster randomized trial | I (ECD + public works programme): 541  I (public works only): 508 | HOME: I (ECD + public works programme) > I (public works only)  OMCI: I (ECD + public works programme) > I (public works only)  MICS: I (ECD + public works programme) > I (public works only)  KAP: ns |  | Dietary diversity: I (ECD + public works programme) > I (public works only)  Shared decision-making: I (ECD + public works programme) > I (public works only)  Illness past week: I (ECD + public works programme) > I (public works only)  WASH: I (ECD + public works programme) > I (public works only)  HSCL-25: I (ECD + public works programme) < I (public works only) |
| Rao et al., 2020 and Chan et al., 2021, Bhutan* (*UNICEF*) | Evaluation study | CCD only: 44  ECCD only: 162  Combined: 49  No CCD or ECCD: 170 | MICS: All groups > No CCD or ECCD | ECDI: All groups > No CCD or ECCD |  |
| Shah et al., 2020, India | Feasibility pilot | I: 47 | MICS: improved from baseline to endline |  |  |
| Shi et al., 2020, China | Cluster-RCT | I: 71  C: 69 |  | ASQ (composite, communication, fine motor): I>C |  |
| Xu et al., 2020, China – ***study protocol*** |  |  |  |  |  |
| †African Population Health Research Centre, 2021, Kenya* (*PATH*) | Cluster-RCT | I: 410  C: 206 | ECD knowledge^1^: ns at mid- and endline  ECD practices: I>C at midline. Ns at endline | ASQ: I>C at midline. Ns at endline |  |
| †Aga Khan University and Aga Khan Foundation, 2021, Kenya* (*Madrasa Early Childhood Programme*) |  |  |  |  |  |
| Aga Khan University and Aga Khan Foundation, 2021, Syria (*AKDN*) |  |  |  |  |  |
| †Aga Khan University and Aga Khan Foundation, 2021, Tanzania* (*Madrasa Early Childhood Programme and Elizabeth Glaser Pediatric AIDS Foundation*) |  |  | HOME, MICS, and RISE: analysis pending |  |  |
| †Aga Khan University and Aga Khan Foundation, 2021, Uganda* (*Madrasa Early Childhood Programme and Save the Children*) |  |  |  |  |  |
| Akhmadi et al., 2021, Indonesia* – ***training evaluation*** |  |  |  |  |  |
| Dovel et al., 2021, Malawi* (*Partners in Hope*) |  |  |  |  |  |
| Jensen et al., 2021, Rwanda | Stratified cluster randomized trial | I (ECD + public works programme): 559  I (public works only): 525 |  | ASQ Communication: I (ECD + public works programme) > I (public works only)  ASQ Fine motor: I (ECD + public works programme) > I (public works only)  ASQ Gross motor: I (ECD + public works programme) > I (public works only)  ASQ Problem-solving: I (ECD + public works programme) > I (public works only)  ASQ Social-emotional: I (ECD + public works programme) > I (public works only)  MDAT Gross motor and language: ns |  |
| Jeong et al., 2021, and Bliznashka et al., 2022 Mozambique* (*PATH*) – ***qualitative evaluation*** |  |  |  |  |  |
| UNICEF 2021, Belize and Anguilla* (*UNICEF and PAHO/WHO*) |  |  |  |  |  |
| UNICEF 2021, the Dominican Republic* (*UNICEF and PAHO/WHO*) |  |  |  |  |  |
| UNICEF 2021, El Salvador* (*UNICEF and PAHO/WHO*) |  |  |  |  |  |
| UNICEF 2021, Peru* (*UNICEF and PAHO/WHO*) |  |  |  |  |  |
| †UNICEF 2021, Tunisia, Iran, Egypt* (*UNICEF*) |  |  |  |  |  |
| †Antelman et al., 2022, Tanzania (*Elizabeth Glaser Pediatric AIDS Foundation*) | Non-randomized pre-post evaluation | I (CCD - radio + home visit/clinic session): 520  No I (Radio-only): 484 | ECD knowledge: I (CCD) > I (radio-only)  MICS (stimulation practices): I (CCD) > I (radio-only)  MICS (father engagement): I (CCD) > I (radio-only)  Responsive care: ns |  | Household environment risk: ns  PSI: I (CCD) < I (radio-only) |
| Bahari Gharehgoz et al., 2022, Iran* | Non-randomized pre-post evaluation | I: 25  No I: 25 | SEAM: I > No I  Maternal Caregiving Quality Scale: I > No I |  |  |
| Bemanalizadeh et al., 2022, Iran* | RCT | I: 80  C: 101 |  | Bayley (cognitive, language, motor): ns  CBCL (attention, anxiety, developmental problems): I < C  CBCL (other sub-scales); ns |  |
| †Ummeed 2022, India (*Ummeed*) |  |  |  |  |  |
| Jeong et al., unpublished manuscript, Tanzania* (*Global Communities*) | Cluster-RCT | I (nutrition, mothers): 162  I (nutrition, couples): 166  I (CCD + nutrition, mothers): 166  I (CCD + nutrition, couples): 154  C: 167 | Maternal FCI: I (CCD + nutrition) > I (nutrition)  Maternal and paternal parenting knowledge, paternal FCI, maternal and paternal MICS: ns | Bayley (cognitive, receptive language): I (CCD + nutrition) > I (nutrition)  Bayley (expressive language, gross motor, fine motor): ns | Paternal PSI (parenting distress subscale): I (CCD + nutrition) < I (nutrition)  Maternal and paternal CRS and SRQ-20, maternal PSI (parenting distress subscale): ns |
| †McHenry et al., unpublished manuscript, Kenya* | Randomized pilot | I (CCD then standard care): 16  I (standard care then CCD): 15 | HOME: ns | Bayley (cognitive, language, motor): ns | PHQ-9: ns |

Note: Missing information (i.e., empty cells) indicates that data were not clearly provided

†Extracted data were supplemented by information provided in key informant interview(s)

*CCD-based service

ASQ = Ages and Stages Questionnaire. C = control group. CBCL: Child Behaviour Checklist. CRS = Co-parenting Relationship Scale-short form. ECD = early childhood development. ECCD = Early Childhood Care and Development. ECDI = Early Childhood Development Index. FCI = Family Care Indicators. HOME = Home Observation Measurement of the Environment. HSCL-25: Hopkins Symptom Checklist-25. I = intervention group. KAP = Knowledge of, attitudes toward, and practice of child rearing. MDAT = Malawi Development Assessment Tool. MICS = Multiple Indicator Cluster Survey. Ns = non-significant. OMCI = Observation for Mother-Child Interactions. PHQ-9: Patient Health Questionnaire-9. PSI = Parenting Stress Index. RCT = randomized controlled trial. SEAM = Social-Emotional Assessment/Evaluation Measure. SRQ-20 = 20-item Self-Reporting Questionnaire.

^1^African Population Health Research Center, 2021 – the specific measures used to assess caregiver knowledge and ECD practices were not indicated in this report. Authors indicate that “caregivers’ ECD knowledge and child-rearing practices were quantified using a standard set of questions regarding each construct”

^2^Jeong et al., unpublished manuscript – for simplicity, only results comparing the bundled CCD and nutrition intervention arms (mother-only and couples) to the nutrition arms (mother-only and couples) are presented here

**References**

African Population and Health Research Centre. (2021). Evaluation of the feasibility and effectiveness of a health facility-based early childhood development (ECD) intervention in Siaya

County, Kenya: Summary Report.

Aga Khan University & Aga Khan Foundation. (2021a). Integrating Playful Parenting Interventions in Government Systems: Experiences of Stakeholders in Implementing Care for Child

Development in Uganda.

Aga Khan University & Aga Khan Foundation. (2021b). The Role of the Health Sector in Advancing Nurturing Care: Aga Khan Development Network Syria’s Implementation of Care for

Child Development.

Aga Khan University & Aga Khan Foundation. (2021c). The Role of Civil Society and Partnerships in Advancing Nurturing Care: Madrasa Early Childhood Programme Zanzibar and the

Elizabeth Glaser Pediatric AIDS Foundation’s Implementation of Care for Child Development.

Aga Khan University & Aga Khan Foundation. (2021d). Leveraging the Community Health Strategy and Multi-Sectoral Partnerships Towards Scaling Up Nurturing Care in Rural

Communities: Madrasa Early Childhood Programme’s Implementation of Care for Child Development in Kenya.

Akhmadi., Sunartini., Haryanti, F., Madyaningrum, E., & Sitaresmi, M. N. (2021). Effect of care for child development training on cadres’ knowledge, attitude, and efficacy in Yogyakarta,

Indonesia. *Belitung Nursing Journal, 7*(4), 311-319

Antelman, G., Ferla, J., Gill, M., Heather, H., Komba, T., Abubakar, A., Remes, P., Jahanpour, O., Mariki, M., Mang’engya, M., & van de Ven, R. (2022). Effectiveness of an integrated

community, facility and media-based early child development intervention on caregiver knowledge and behavior: A quasi-experimental evaluation of the Malezi program in Tanzania.

Pre-print article.

Bahari Gharehgoz, A., Heidarabadi, S., Alizadeh, H., & Asgari, M. (2022). Effectiveness of Care for Child Development Program on the Sensitivity and Responsiveness Skills of Mothers. *Iranian Journal of Child Neurology, 16*(1), 51-63.

Barnhart, D. A., Farrar, J., Murray, S. M., Brennan, R. T., Antonaccio, C. M., Sezibera, V., ... & Betancourt, T. S. (2020). Lay-worker delivered home visiting promotes early childhood

development and reduces violence in Rwanda: a randomized pilot. *Journal of Child and Family Studies*, *29*(7), 1804-1817

Bayitondere, S., Biziyaremye, F., Kirk, C. M., Magge, H., Hann, K., Wilson, K., ... & Miller, A. C. (2018). Assessing retention in care after 12 months of the Pediatric Development Clinic

implementation in rural Rwanda: a retrospective cohort study. *BMC pediatrics*, *18*(1), 1-11.

Bemanalizadeh, M., Badihian, N., Khoshhali, M., Badihian, S., Hosseini, N., Purpirali, M., Abadian, M., Yaghini, O, Daniali, S.S., & Kelishadi, R. (2022). Effect of parenting intervention

through “Care for Child Development guideline” on early child development and behaviors: A randomized controlled trial. Pre-print article.

Betancourt, T. S., Jensen, S. K., Barnhart, D. A., Brennan, R. T., Murray, S. M., Yousafzai, A. K., ... & Kamurase, A. (2020). Promoting parent-child relationships and preventing violence via

home-visiting: a pre-post cluster randomised trial among Rwandan families linked to social protection programmes. *BMC public health*, *20*(1), 1-11

Bliznashka, L., Yousafzai, A. K., Asheri, G., Masanja, H., & Sudfeld, C. R. (2021). Effects of a community health worker delivered intervention on maternal depressive symptoms in rural

Tanzania. *Health policy and planning*, *36*(4), 473-483

Bliznashka, L., Ahun, M. N., Velthausz, D., Donco, R., Karuskina-Drivdale, S., Pinto, J., ... & Jeong, J. (2022). Effects of COVID-19 on Child Health Services Utilisation and Delivery in Rural

Mozambique: A Qualitative Study. *Health Policy and Planning*

Chan, S. W., Rao, N., Cohrssen, C., & Richards, B. (2021). Predicting child outcomes in Bhutan: Contributions of parenting support and early childhood education programmes. *Children and*

*Youth Services Review*, *126*, 106051

Dovel, K., Kalande, P., Udedi, E., Temelkovska, T., Hubbard, J., Mbalanga, C., ... & Coates, T. J. (2021). Integrated early childhood development services improve mothers’ experiences with prevention of mother to child transmission (PMTCT) programs in Malawi: a qualitative study. *BMC health services research*, *21*(1), 1-9

Drivdal, S.K., Kwakyu, N., & Mulhanga, F. (2015). A playbox intervention in health facility waiting rooms in Mozambique: Improving caregivers’ knowledge, skills and communication with

health professionals. *International Journal of Birth and Parent Education, 6*(3), 29-32

Engle, P. & Najimidinova, G., Agency of Sociological and Marketing Surveys, and Faromuzova, K. (2011) Care for Development in Three Central Asian Countries: Report of a Process

Evaluation in Tajikistan, Kyrgyz Republic, and Kazakhstan. UNICEF, Geneva, Switzerland.

Ertem, I. O., Atay, G., Bingoler, B. E., Dogan, D. G., Bayhan, A. & Sarica, D. (2006). Promoting child development at sick-child visits: a controlled trial. Pediatrics, 118, 3124-3131.

Ertem, I. O., Pekcici, E. B. B., Gok, C. G., Ozbas, S., Ozcebe, H., & Beyazova, U. (2009). Addressing early childhood development in primary health care: experience from a middle-income

country. *Journal of Developmental & Behavioral Pediatrics*, *30*(4), 319-326

Fisher, J., Tran, T., Luchters, S., Tran, T. D., Hipgrave, D. B., Hanieh, S., ... & Biggs, B. A. (2018). Addressing multiple modifiable risks through structured community-based Learning Clubs to

improve maternal and infant health and infant development in rural Vietnam: protocol for a parallel group cluster randomised controlled trial. *BMJ open*, *8*(7), e023539

Gladstone G, McLinden M, Douglas G, et al. (2017) ‘Maybe I will give some help … maybe not to help the eyes but different help’: an analysis of care and support of children with visual

impairment in community settings in Malawi. *Child: Care, Health and* *Development* *43*(4): 608–620.

Gladstone, M. J., Phuka, J., Thindwa, R., Fatima, C., Chidzalo, K., Gledowe-Ware, S., & Maleta, K. (2018). Care for Child Development in rural Malawi–a model feasibility and pilot study. *Annals of the New York Academy of Sciences*, *1419*(1), 102-119

Holding, P. (2015). A child is one to be raised: An exploration of Care for Child Development, Mali 2015.

Jensen, S. K., Placencio-Castro, M., Murray, S. M., Brennan, R. T., Goshev, S., Farrar, J., ... & Betancourt, T. S. (2021). Effect of a home-visiting parenting program to promote early childhood

development and prevent violence: a cluster-randomized trial in Rwanda. *BMJ* *global health*, *6*(1), e003508

Jeong, J., Bliznashka, L., Ahun, M. N., Karuskina‐Drivdale, S., Picolo, M., Lalwani, T., ... & Yousafzai, A. K. (2021). A pilot to promote early child development within health systems in

Mozambique: a qualitative evaluation. *Annals of the New York Academy of* *Sciences*

Jeong, J., Ahun, M.N., Ambikapathi, R., Gunaratna, N.S., Mapendo, F., Galvin, L., Kieffer, M.P., Mwanyika-Sando, M., Mosha, D., Froese, S.L., Verissimo, C.K., Praygod, G., & Yousafzai,

A.K. (unpublished manuscript). Effects of engaging fathers and bundling parenting and nutrition interventions on early child development and maternal and paternal parenting in rural Tanzania: A five-arm cluster-randomized controlled trial

Jin, X., Sun, Y., Jiang, F., Ma, J., Morgan, C. & Shen, X. (2007) Care for Development intervention in rural China: a prospective followup study. Journal of Developmental and Behavioral

Pediatrics, l28, 213–218.

Jones, L. (2012). Report on an ethnographic study of child rearing in Cabo Delgado province, Mozambique. Aga Khan Foundation.

Lingam, R., Gupta, P., Zafar, S., Hill, Z., Yousafzai, A., Iyengar, S., ... & Kirkwood, B. (2014). Understanding care and feeding practices: building blocks for a sustainable intervention in India

and Pakistan. *Annals of the New York Academy of Sciences*, *1308*(1), 204-217

Lynch, P., Gladstone, M., McLinden, M., Douglas, G., Jolley, E., Schmidt, E., & Chimoyo, J. (2018). ‘I have learnt to love the child and give opportunities to play with peers’: A feasibility study

of the training program to support parents of young children with visual impairment in Malawi. *Journal of Early Childhood Research*, *16*(2), 210-225.

McHenry, M.S., Alex, B., Roose, A., Raciti, C., Oyungu, E., Ombitsa, A.R., Cherop, C., Kaniaru, B., Cherop, C., John, C.C., & Vreeman, R.C. (unpublished manuscript). Acceptability and

feasibility of a group-based intervention to improve outcomes for children at risk for developmental delays: A piloted randomized trial

Rao, M., Cohrssen, C., Chan, S., & Richards, B. (2020). An evaluation of the early childhood care and development programme in Bhutan. Ministry of Education Bhutan and UNICEF Bhutan

Country Office

Rockers, P. C., Fink, G., Zanolini, A., Banda, B., Biemba, G., Sullivan, C., ... & Hamer, D. H. (2016). Impact of a community-based package of interventions on child development in Zambia: a

cluster-randomised controlled trial. *BMJ Global Health*, *1*(3), e000104

Rochat, T. J., Dube, S., Herbst, K., Hoegfeldt, C. A., Redinger, S., Khoza, T., Bland, R.M., Richter, L., Linsell, L., Desmond, C., Yousafzai, A.K., Craske, M., Juszczak, E., Abas, M., Edwards,

T., Ekers, D., & Stein, A. (2021). An evaluation of a combined psychological and parenting intervention for HIV-positive women depressed in the perinatal period, to enhance child

development and reduce maternal depression: study protocol for the Insika Yomama cluster randomised controlled trial. *Trials*, *22*(1), 1-23.

Rosales, A., Sargsyan, V., Abelyan, K., Hovhannesyan, A., Ter-Abrahanyan, K., Jillson, K. Q., & Cherian, D. (2019). Behavior change communication model enhancing parental practices for

improved early childhood growth and development outcomes in rural Armenia–A quasi-experimental study. *Preventive medicine reports*, *14*, 100820

Shah, R., Gustafson, E., Dhaded, S., Herekar, V., Metgud, D., Mastiholi, S., ... & Atkins, M. (2020). Integrating an Adapted, Low-Intensity Program to Promote Early Childhood Development in

Routine Health Visits in Rural India: A Feasibility Study. *Journal of* *Developmental & Behavioral Pediatrics*, *41*(4), 281-288.

Shi, H., Li, X., Fang, H., Zhang, J., & Wang, X. (2020). The Effectiveness and Cost-effectiveness of a Parenting Intervention Integrated with Primary Health Care on Early Childhood

Development: a Cluster-Randomized Controlled Trial. *Prevention Science*, *21*(5), 661-671

SPRING Trial Team. Personal communication of SPRING Trial in India and Pakistan from key informant interview in February 2022.

Sudfeld, C. R., Bliznashka, L., Ashery, G., Yousafzai, A. K., & Masanja, H. (2019). Effect of a community health worker delivered health, nutrition and responsive stimulation package and

conditional cash transfers on child development and growth in rural Tanzania: protocol for a cluster-randomized trial. *BMC Public Health*, *19*(1), 1-7

Sudfeld, C.R., Bliznashka, L., Ashery, G., Yousafzai, A.K., & Masanja, H. (2021). Effect of a home-based health, nutrition and responsive stimulation intervention and conditional cash transfers

on child development and growth: a cluster-randomised controlled trial in Tanzania. *BMJ Global Health*, *6*:e005086

Thorne, C. (2014). Care for Child Development in 5 Countries: Tanzania, Kenya, Uganda, Kyrgyzstan, and Tajikistan. A multi-country review of C4CD pilots across the Aga Khan Development

Network. Aga Khan Foundation.

Ummeed (2022). Early Childhood Development and Disability Programs: Impact Study Report 2015-21. Ummeed, India.

UNICEF (2019). Care for Child Development Case Study: The experience of Paraguay. United Nations Children’s Fund, New York, USA.

UNICEF (2021a). UNICEF Regional Guidance for Middle East & North Africa on Care for Child Development In-Country Roll-Out. United Nations Children’s Fund Middle East and North Africa

Regional Office, Amman.

UNICEF (2021b). Care for Child Development Case Study: The experience of the Dominican Republic. United Nations Children’s Fund Latin America and Caribbean Regional Office, Panama.

UNICEF (2021c). Care for Child Development Case Study: The experience of El Salvador. United Nations Children’s Fund Latin America and Caribbean Regional Office, Panama.

UNICEF (2021d). Care for Child Development Case Study: The experience of Peru. United Nations Children’s Fund Latin America and Caribbean Regional Office, Panama.

UNICEF (2021e). Care for Child Development Case Study: The experience of Belize. United Nations Children’s Fund Latin America and Caribbean Regional Office, Panama.

World Vision International (2017). Evidence four change: Go Baby Go! World Vision Middle East, Eastern Europe Regional Office, Cyprus

Xu, M., Liu, A., Zhao, C., Fang, H., Huang, X., Berman, S., & Guan, H. (2020). Group-based intervention to improve developmental status among children age 6–18 months in rural Shanxi

province, China: a study protocol for a cluster randomised controlled trial. *BMJ* *open*, *10*(10), e037156

Yousafzai, A. K., Rasheed, M. A., Rizvi, A., Armstrong, R. & Bhutta, Z. A. (2014) Effect of integrated responsive stimulation and nutrition interventions in the Lady Health Worker program in

Pakistan on child development, growth, and health outcomes: a cluster randomised factorial effectiveness trial. Lancet, 384, 1282–1293.

Yousafzai, A. K., Rasheed, M. A., Rizvi, A., Armstrong, R., & Bhutta, Z. A. (2015). Parenting skills and emotional availability: an RCT. *Pediatrics*, *135*(5), e1247-e1257

Yousafzai, A. K., Rasheed, M. A., & Siyal, S. (2018). Integration of parenting and nutrition interventions in a community health program in Pakistan: an implementation evaluation. *Annals of the*

*New York Academy of Sciences*, *1419*(1), 160-178

Zhou, S., Zhao, C., Huang, X., Li, Z., Ye, R., Shi, H., ... & Scherpbier, R. W. (2019). The effect of a community-based, integrated and nurturing care intervention on early childhood development

in rural China. *Public Health*, *167*, 125-135
